# Digital Exclusion and Cognitive Function in Elderly Populations in Developing Countries: Insights Derived From 2 Longitudinal Cohort Studies

**DOI:** 10.2196/56636

**Published:** 2024-11-15

**Authors:** Sainan Duan, Dongxu Chen, Jinping Wang, Mohammed Sharooq Paramboor, Zhen Xia, Wanting Xu, Kun Han, Tao Zhu, Xiaoqin Jiang

**Affiliations:** 1 Department of Anesthesiology West China Second University Hospital Sichuan University Chengdu China; 2 Key Laboratory of Birth Defects and Related Diseases of Women and Children (Sichuan University) Ministry of Education Chengdu China; 3 Department of Anesthesiology West China Hospital Sichuan University Chengdu China; 4 The Research Units of West China (2018RU012)-Chinese Academy of Medical Sciences West China Hospital Sichuan University Chengdu China; 5 West China School of Medicine Sichuan University Chengdu China; 6 Department of Anesthesiology Shengli Oilfield Central Hospital Dongying China

**Keywords:** digital exclusion, cognition, cohort study, developing country, China Health and Retirement Longitudinal Study, CHARLS, MHAS, cognitive decline

## Abstract

**Background:**

Cognition disorders not only lead to adverse health consequences but also contribute to a range of socioeconomic challenges and diminished capacity for performing routine daily activities. In the digital era, understanding the impact of digital exclusion on cognitive function is crucial, especially in developing countries.

**Objective:**

This study aimed to evaluate the association between digital exclusion and cognitive function among elderly populations in developing countries.

**Methods:**

Using data from CHARLS (China Health and Retirement Longitudinal Study) from 2011 to 2020 and MHAS (Mexican Health & Aging Study) from 2012 to 2021, we defined digital exclusion as self-reported absence from the internet. Cognitive function was assessed through 5 tests: orientation, immediate verbal recall, delayed verbal recall, serial 7s, and figure recall. Cognitive function was assessed in 2 categories: worse cognition (a categorical variable that classifies cognition as either better or worse compared to the entire cohort population) and cognitive scores (a continuous variable representing raw cognitive scores across multiple follow-up waves). Logistic regression analyses and generalized estimating equation (GEE) analyses were used to examine the relationship between cognitive function and digital exclusion, adjusting for potential confounders, including demographics, lifestyle factors, history of chronic diseases, basic activities of daily living (BADL) disability, instrumental activities of daily living (IADL) disability, and basic cognitive abilities.

**Results:**

After excluding participants with probable cognitive impairment at baseline and those who did not have a complete cognitive assessment in any given year (ie, all tests in the cognitive assessment must be completed in any follow-up wave), a total of 24,065 participants in CHARLS (n=11,505, 47.81%) and MHAS (n=12,560, 52.19%) were included. Of these, 96.78% (n=11,135) participants in CHARLS and 70.02% (n=8795) in MHAS experienced digital exclusion. Adjusted logistic regression analyses revealed that individuals with digital exclusion were more likely to exhibit worse cognitive performance in both CHARLS (odds ratio [OR] 2.04, 95% CI 1.42-2.99; *P*<.001) and MHAS (OR 1.40, 95% CI 1.26-1.55; *P*<.001). Gender and age did not significantly modify the relationship between digital exclusion and worse cognition (intervention *P*>.05). The fully adjusted mean differences in global cognitive scores between the 2 groups were 0.98 (95% CI 0.70-1.28; *P*<.001) in CHARLS and 0.50 (95% CI 0.40-0.59; *P*<.001) in MHAS.

**Conclusions:**

A substantial proportion of older adults, particularly in China, remain excluded from internet access. Our study examined longitudinal changes in cognitive scores and performed cross-sectional comparisons using Z-score standardization. The findings suggest that digital exclusion is linked to an increased risk of cognitive decline among older adults in developing countries. Promoting internet access may help mitigate this risk and support better cognitive health in these populations.

## Introduction

The worldwide phenomenon of population aging presents significant health and socioeconomic challenges. Notably, China, home to the world’s largest older adult population, is projected to see over 479 million individuals aged 60 years and above by 2050 [1]. The prevalence of dementia among older adults in China is anticipated to escalate to 18 million by 2030 [2]. In Western developing countries, such as Mexico, the prevalence of dementia among people over 65 years old (7.1%-11.5%) is increasing compared to Europe and the United States [3]. These trends underscore the urgent need for effective public health strategies focusing on dementia prevention and treatment. Cognitive decline, characterized by a deterioration in memory and other cognitive abilities, manifests as a noticeable reduction in cognitive performance compared to previous levels [4], poses substantial socioeconomic challenges, and affects the ability to perform daily activities. It is further exacerbated by factors such as chronic conditions (eg, hypertension or hearing impairment) [5]. However, significant gaps remain in understanding modifiable risk factors and their potential role in the prevention or decelerating cognitive decline.

With technological advancements, the internet has become an essential digital platform for communication, information access [6], and medical consultation [7]. However, digital exclusion—defined as the lack of access to information and communications technologies, including the internet [8]—remains prevalent. In more prosperous countries with well-established market economies, internet usage rates are high [9], but in some developing countries, the proportion of internet users is significantly lower, particularly among the elderly. For example, digital exclusion among the elderly reaches 97% in China and 66% in Mexico, significantly exceeding 25%-65% typical in other nations [10]. The association between mental health and internet use has been extensively explored [11-13], although the association remains unclear. Additionally, research indicated that middle-aged and older adults who use the internet have a lower risk of developing chronic diseases compared to those who do not use the internet [14]. Digital exclusion among older adults is associated with functional dependence, as evidenced by 5 longitudinal cohort studies [10]. Previous studies have suggested that in developed countries, such as England [15], Australia [16], and the United States [17], internet access can influence cognitive function in older individuals. The potential mechanisms for explaining the association between digital exclusion and cognition could be explained as follows: the internet serves as a critical tool for the elderly population, enabling access to up-to-date health management information, medication purchases, and health devices procurement [18]. It also facilitates timely consultations with health care professionals and real-time data monitoring, which are essential for effective health management [19,20]. Additionally, vulnerable digital excluders, such as those with lower levels of social communication with colleagues or family members, are more likely to experience poor health status compared to those who are not in a vulnerable position. This observation aligns with studies highlighting that internet engagement promotes social activities that can alleviate loneliness and enhance overall health [21]. Thus, the internet has emerged as an alternative means to real-life interactions, helping reduce social isolation and enhance the quality of life for older adults [22]. However, there is limited research on the association between digital exclusion and cognitive performance among the elderly in developing countries. Liu et al [23] demonstrated a correlation between digital exclusion and cognitive impairment in a cross-sectional study of 10,325 people in China, but no causal relationships could be established. Through cross-sectional and longitudinal data, Li et al [24] revealed that internet use can help prevent the contraction of the pallidum and thus mitigate the decline of cognitive function in elderly Chinese individuals. However, this study only conducted a 1-year follow-up [24]. Jin et al [25] conducted a similar study that was still limited to China. The methodological limitations of many existing studies encompass varied deﬁnitions of cognitive decline [23-25], cross-sectional design [23,24], or geographic limitations [23-25], as well as insufﬁcient analysis of risk factors in developing countries, thus obscuring the actual role of internet use on cognitive decline risk. Similar attempts are also needed to elucidate the role of digital exclusion in these regions.

CHARLS (China Health and Retirement Longitudinal Study) and MHAS (Mexican Health & Aging Study) cohorts, comprising large, prospective longitudinal data sets with a wealth of variables and extensive follow-up information, represented ideal study populations for this study. Both China and Mexico, as developing countries, exhibit high rates of digital exclusion, further underscoring the relevance of these cohorts for examining the impacts of this association. In addition, differences in regional, demographic, and educational characteristics between the 2 cohorts not only allow for a robust verification of study results but also enhance their generalizability. Thus, we used comprehensive data from CHARLS and MHAS to examine the impact of digital exclusion on cognitive function among older adults in developing countries. Furthermore, we specifically incorporated the latest CHARLS Wave 5 National Follow-up Survey data of 2020, released on November 16, 2023.

## Methods

### Ethical Considerations

CHARLS was approved by Peking University (IRB00001052-11015), and MHAS was approved by the University of Texas Medical Branch in the United States, the Instituto Nacional de Estadística y Geografía (INEGI), and the Instituto Nacional de Salud Pública (INSP) in Mexico. Written informed consent was obtained from all participants prior to the survey. This study adhered to the ethical guidelines outlined in the Declaration of Helsinki. There was no requirement for additional ethics approval for approved data users in CHARLS and MHAS [26,27].

### Data Source and Population

CHARLS, a comprehensive longitudinal survey, targets individuals aged 45 years or older and their spouses across 150 counties/districts and 450 villages/urban areas in 28 provinces of China, including assessments of the social, economic, and health conditions of community residents [26]. MHAS is a national longitudinal study focusing on adults aged 50 years or above in Mexico, aimed at prospectively evaluating the impact of disease on health, function, and mortality among Mexican adults [27]. Data for this study were sourced from the publicly accessible CHARLS and MHAS databases. This study incorporated data from 5 waves of CHARLS (2011 wave 1, 2013 wave 2, 2015 wave 3, 2018 wave 4, 2020 wave 5) and 4 waves from MHAS (2012 wave 3, 2015 wave 4, 2018 wave 5, and 2021 wave 6).

Participants with memory-related disorders, brain damage, or stroke at baseline (2011 wave 1 in CHARLS, 2012 wave 3 in MHAS) were excluded. Also excluded were those lacking digital evaluation at baseline or those without a single complete cognitive assessment in subsequent waves. Finally, the analyses included 11,505 eligible participants from CHARLS and 12,560 from MHAS.

### Ascertainment of Digital Exclusion

Data regarding digital exclusion were obtained via self-completed questionnaires. In CHARLS, digital exclusion was assessed with question DA056: “Have you used the internet in the past month?” For MHAS, where individual internet usage was not directly assessed, an alternative question (J18), “In this house do you have Internet?” was used to infer digital exclusion [10]. A response of no to both questions was categorized as evidence of digital exclusion, whereas a response of yes indicated inclusion.

### Assessment of Cognitive Function

Cognitive functional status was assessed using 2 methods: categorical (based on the standardized *Z*-score, categorizing cognition as either better or worse cognition compared to the entire cohort population) and continuous (cognitive raw scores in multiple follow-up waves). Cognitive scores included assessments across 5 tests: orientation, immediate verbal recall, delayed verbal recall, serial 7s, and figure recall [28,29]. Notably, the serial 7s component was unavailable in the MHAS cohort. Orientation assessment in CHARLS covered the year, month, day, day of the week, and seasonal, for a total of 5 points. In MHAS, the orientation assessment excluded the day of the week and season. Immediate and delayed verbal recall tasks involved recalling unrelated words, with 1 point given for each word recalled. The maximum score for immediate verbal recall was 10 points in CHARLS and 8 in MHAS, while delayed verbal recall had a ceiling of 10 points in CHARLS and 8 in MHAS. Figure recall involved participants replicating a previously given graph, scoring 1 point in CHARLS and up to 6 points in MHAS. The serial 7s task required counting backward from 100 by 7 five times, with each correct response earning 1 point. Overall, the global cognitive scores ranged from 0 to 31 in CHARLS and from 0 to 25 in MHAS, with higher scores indicating superior cognitive performance [30]. Detailed methodologies of cognitive function assessments in both CHARLS and MHAS are documented in Tables S1 and S2 in Multimedia Appendix 1, respectively.

In this study, worse cognition was used as a proxy for assessing cognitive function in comparison to others at the follow-up endpoint. Worse cognition was accessed in 2 steps. First, the cognitive score was obtained from all follow-up waves, and then the raw score was standardized to the baseline to obtain the *Z*-score [30]. For instance, in CHARLS, the mean (SD) of global cognitive scores at baseline was 14.91 (1.35). Next, the *Z*-score was calculated as (last complete raw cognitive score – 14.91)/1.35, with positive *Z*-values indicating better cognitive ability than the mean population and negative *Z*-values suggesting worse cognition [29]. Worse cognition was used to assess performance in comparison to other participants, and the methodology for this assessment was based on previous published studies [29,30].

### Covariates

Covariates were identiﬁed through literature reviews and included demographics, lifestyle, history of chronic diseases, basic activities of daily living (BADL) disability, instrumental activities of daily living (IADL) disability, and anthropometric measures. Demographics included age, gender, marital status (single, married or cohabiting, and divorced or widowed), education level, and residence (rural or urban). Education levels were divided into lower secondary (illiteracy, primary school, and junior high schools), upper secondary and vocational training, and tertiary (bachelor’s degree or above). Lifestyle factors assessed were smoking and drinking status. A history of chronic diseases encompassed hypertension, diabetes, dyslipidemia, heart disease, respiratory illness, liver disease, kidney disease, digestive disease, arthritis, rheumatism, and cancer. BADL were assessed using the Katz scale with 6 items: continence, dressing, bathing, feeding, transferring, and going to the toilet [31]. According to Lawton and Brody [32], IADL include housekeeping, preparing hot meals, shopping, managing money, taking medications, and using the telephone. BADL disability and IADL disability were defined as having difficulties in doing any item in BADL and IADL, respectively [33]. Anthropometric measures included the hand grip test (kg) and waist circumference (cm). The BMI was calculated as the measure of body weight in kilograms divided by the measure of body height in meters squared. Cognitive scores at baseline were considered candidate covariates in statistical analysis. Additional information about the covariates in CHARLS and MHAS is presented in Tables S3 and S4 in Multimedia Appendix 1, respectively.

### Statistical Analysis

Categorical variables were described as percentages, with missing values indicated by “unknown,” and continuous variables were expressed as mean (SD) or median (IQR; 25th-75th percentiles, Q1-Q3). Continuous variables were compared between groups using the Wilcoxon rank-sum test, and categorical variables were compared using the Pearson chi-squared test or the Fisher exact test. To identify covariates with high multicollinearity, a generalized variance inflation factor (GVIF) analysis was conducted [34-36], with adjusted generalized standard error inflation factor (aGSIF) values below 1.6 deemed acceptable [36]. We assessed the associations between digital exclusion and worse cognition using odds ratios (ORs), with 95% CIs derived from logistic regression models, partly (models 1-3) or fully (model 4), adjusted for age at baseline (continuous variable), gender (male or female), BMI group (≤24 kg/m^2^ or >24 kg/m^2^), marital status (single, married, or divorced), education level (lower secondary, upper secondary, or tertiary), residence (rural or urban), smoking status (current or never), drinking status (ever, current, or never), BADL and IADL disabilities (yes or no), global cognitive scores at baseline (continuous variable), and chronic diseases (yes or no). Furthermore, to detect the modification effect of age and sex on the studied associations, we performed subgroup analyses by age group (<50, 50-59, or >59 years) and sex (male or female).

Generalized estimating equations (GEEs) extend the generalized linear model to allow further longitudinal data analysis [37]. We appointed exchangeable structures as working correlation structures and analyzed raw cognitive scores (including the scores and total scores for each section of the cognitive tests) with GEE models [38] to evaluate the differences in the changes between digital exclusive and inclusive cognitive scores of follow-up waves [39]. In addition, the interaction of the time variable with cognitive scores was conducted to examine whether cognitive scores vary by digital exclusion [40].

R software (v4.3.0; R Core Team) was used for all statistical data analyses. The *geepack* package was used to perform GEE analysis. Two-tailed *P*<.05 was considered statistically significant.

## Results

### Baseline Characteristics of Participants

The final analysis included a total of 24,065 participants. In CHARLS, 3826 (15.9%) samples were excluded due to incomplete follow-up data, resulting in a final data set of 11,505 (47.81%) observations. In MHAS, 2724 (11.32%) samples were excluded due to incomplete follow-up data, leaving a final data set of 12,560 (52.19%) observations, as detailed in Tables 1 and 2 and in Figure S1 in Multimedia Appendix 1. The missing data for the covariate are referenced in Figure S2 in Multimedia Appendix 1. The mean age of participants was 57.28 years (SD 8.76) in CHARLS and 62.43 years (SD 10.01) in MHAS. A significant divergence was observed between the 2 cohorts in various measured parameters. Participants in CHARLS typically exhibited poorer health outcomes, as evidenced by higher comorbidity rates and more prevalent unhealthy lifestyles. Conversely, the MHAS cohort, characterized by an older age profile and a higher BMI, demonstrated increased challenges in BADL disability.

**Table 1:** Descriptive statistics in CHARLS^a^ and MHAS^b^.

Characteristics			CHARLS (n=11,505)		MHAS (n=12,560)
Age (years), mean (SD)			57.28 (8.76)		62.43 (10.01)
**Gender, n (%)**					
	Male	5666 (49.25)		5170 (41.16)	
	Female	5839 (50.75)		7390 (58.84)	
BMI (kg/m^2^), mean (SD)			23.93 (10.76)		29.04 (5.26)
**Marital status, n (%)**					
	Single	74 (0.64)		554 (4.41)	
	Married or cohabiting	10421 (90.58)		9110 (72.53)	
	Divorced or widowed	1010 (8.78)		2896 (23.06)	
**Education, n (%)**					
	Lower secondary	9811 (85.28)		9895 (78.78)	
	Upper secondary and vocational training	311 (2.70)		1513 (12.05)	
	Tertiary	1379 (11.99)		1099 (8.75)	
	Unknown	4 (0.03)		53 (0.42)	
**Region of residence, n (%)**					
	Rural	5374 (46.71)		—^c^	
	Town	4106 (35.69)		—	
	Unknown	2025 (17.60)		—	
**Smoking status, n (%)**					
	Current	3612 (31.40)		1552 (12.36)	
	Never	6944 (60.36)		8002 (63.71)	
	Ever	945 (8.21)		3005 (23.93)	
	Unknown	4 (0.03)		1 (0.01)	
**Drinking status, n (%)**					
	Current	3977 (34.57)		3060 (24.36)	
	Never	7526 (65.42)		9497 (75.61)	
	Unknown	2 (0.02)		3 (0.02)	
**Chronic disease, n (%)**					
	Hypertension	2560 (22.25)		5211 (41.49)	
	Diabetes	608 (5.28)		2630 (20.94)	
	Dyslipidemia	1061 (9.22)		—	
	Heart disease	1316 (11.44)		371 (2.95)	
	Respiratory illness	1203 (10.46)		661 (5.26)	
	Liver disease	456 (3.96)		—	
	Kidney disease	714 (6.21)		—	
	Digestive disease	2567 (22.31)		—	
	Arthritis or rheumatism	3696 (32.13)		1630 (12.98)	
	Cancer	101 (0.88)		229 (1.82)	
**BADL^d^ disability, n (%)**					
	Yes	670 (5.82)		1700 (13.54)	
	No	4670 (40.59)		5222 (41.58)	
	Unknown	6165 (53.59)		5638 (44.89)	
**IADL^e^ disability, n (%)**					
	Yes	844 (7.34)		844 (6.72)	
	No	10655 (92.61)		10512 (83.69)	
	Unknown	6 (0.05)		1204 (9.59)	
**Hand grip test (k** **g)**					
	Mean (SD)	30.93 (10.05)		25.66 (8.65)	
	Median (IQR; quartiles 1-3)	30.00 (24.00-38.00)		25.00 (20.00-31.00)	
**Waist circumference, cm**					
	Mean (SD)	84.52 (12.58)		98.29 (12.46)	
	Median (IQR; quartiles 1-3)	85.00 (78.00-92.00)		97.70 (90.00-105.80)	

^a^CHARLS: China Health and Retirement Longitudinal Study.

^b^MHAS: Mexican Health & Aging Study.

^c^Not applicable.

^d^BADL: basic activities of daily living. BADL disability was defined as having difficulties in continence, dressing, bathing, feeding, transferring, or going to the toilet.

^e^IADL: instrumental activities of daily living. IADL disability was defined as having difficulties in housekeeping, preparing hot meals, shopping, managing money, or taking medications.

**Table 2:** Baseline cognitive function in CHARLS^a^ and MHAS^b,c^.

Cognitive function		CHARLS (n=11,505)	MHAS (n=12,560)
**Global cognition**			
	Mean (SD)	16.54 (4.53)	17.74 (3.45)
	Median (IQR; quartiles 1-3)	17.00 (13.00-20.00)	18.33 (15.67-20.33)
**Orientation**			
	Mean (SD)	4.32 (0.93)	2.55 (0.75)
	Median (IQR; quartiles 1-3)	5.00 (4.00-5.00)	3.00 (2.00-3.00)
**Immediate verbal recall**			
	Mean (SD)	4.30 (1.64)	4.88 (1.18)
	Median (IQR; quartiles 1-3)	4.00 (3.00-5.00)	5.00 (4.00-5.67)
**Delayed verbal recall**			
	Mean (SD)	3.37 (1.94)	4.60 (1.98)
	Median (IQR; quartiles 1-3)	3.00 (2.00-5.00)	5.00 (4.00-5.67)
**Serial 7s**			
	Mean (SD)	3.30 (1.82)	—^d^
	Median (IQR; quartiles 1-3)	4.00 (1.00-5.00)	—
**Figure recall**			
	Mean (SD)	0.72 (0.45)	5.59 (0.99)
	Median (IQR; quartiles 1-3)	1.00 (0.00-1.00)	6.00 (6.00-6.00)

^a^CHARLS: China Health and Retirement Longitudinal Study.

^b^MHAS: Mexican Health & Aging Study.

^c^Scores are presented in different ranges due to different assessment methods. CHARLS: global cognition (0-31 points), orientation (0-5 points), immediate verbal recall (0-10 points), delayed verbal recall (0-10 points), serial 7s (0-5 points), and figure recall (0-1 points). MHAS: global cognition (0-25 points), orientation (0-3 points), immediate verbal recall (0-8 points), delayed verbal recall (0-8 points), and figure recall (0-6 points).

^d^Not applicable.

A high prevalence of digital exclusion was reported by 96.78% (n=11,135) participants in CHARLS and 70.02% (n=8795) in MHAS. The characteristics of participants, divided into digitally excluded and included groups, are summarized in Table S5 in Multimedia Appendix 1. Significant differences were observed in several demographic and health-related parameters between these groups in both cohorts. Briefly, participants experiencing digital exclusion tended to be older, had lower levels of education, and were more likely to suffer from disabilities related to daily living activities in both cohorts.

### Association Between Digital Exclusion and Worse Cognition

**Figure 1:**
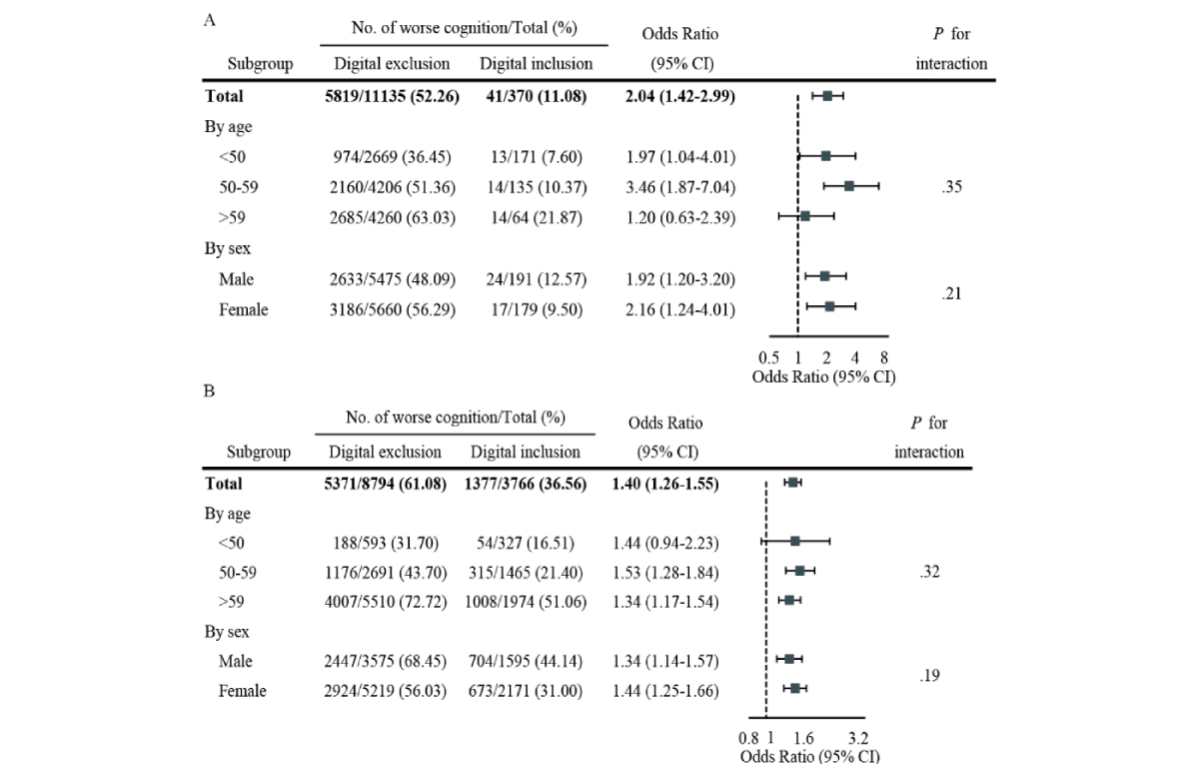
Subgroup analysis of digital exclusion and worse cognition by age group and sex in (A) CHARLS and (B) MHAS. Values are adjusted for age, gender, BMI group, marital status, education level, residence, smoking status, drinking status, BADL and IADL disabilities, chronic diseases, and baseline cognitive scores. BADL: basic activities of daily living; CHARLS: China Health and Retirement Longitudinal Study; IADL: instrumental activities of daily living; MHAS: Mexican Health & Aging Study; OR: odds ratio.

Table 3 presents the relationship between digital exclusion and cognitive performance. When categorizing cognitive function based on overall cognitive scores (indicative of worse cognition), a higher incidence of worse cognition was observed among digitally excluded participants (CHARLS: n=5819, 52.26%, vs n=41, 11.08%; MHAS: n=5371, 61.08%, vs n=1377, 35.56%). In CHARLS, the age-adjusted, sex-adjusted, BMI group–adjusted, and baseline cognitive score–adjusted OR for worse cognition in digitally excluded participants was 3.37 (95% CI 2.39-4.89), as shown in model 1 in Table S6 in Multimedia Appendix 1). This OR reduced to 2.06 (95% CI 1.44-3.03) with further adjustments for additional confounders (models 2 and 3, Table S6 in Multimedia Appendix 1), and the fully adjusted OR (model 4, Table S6 in Multimedia Appendix 1) was 2.04 (95% CI 1.42-2.99). In MHAS, the association remained statistically significant (OR 1.40, 95% CI 1.26-1.55), with similar ORs observed across models with varying levels of adjustment (Table S6 in Multimedia Appendix 1). The elevated risk of worse cognition in individuals with prepandemic psychiatric disorders did not differ by age and sex in both CHARLS and MHAS (Figure 1). Table S7 in Multimedia Appendix 1 shows that all aGSIF values were close to 1 in CHARLS and MHAS, indicating the absence of multicollinearity among confounders in both cohorts.

**Table 3:** Cognition outcome in CHARLS^a^ and MHAS^b^ stratified by exposure.^c^

Cognition			CHARLS (n=11,505)				MHAS (n=12,560)		
			Digital exclusion (n=11,135)		Digital inclusion (n=370)		Digital exclusion (n=8794)		Digital inclusion (n=3766)
Worse cognition (measured by category), n (%)			5819 (52.26)		41 (11.08)		5371 (61.08)		1377 (36.56)
**Global cognition (measured by scores); CHARLS *P*<.001, MHAS *P*<.001**									
	Mean (SD)	15.61 (5.88)		21.54 (3.75)		16.27 (3.94)		18.35 (3.31)	
	Median (IQR; quartiles 1-3)	16.00 (12.00-20.00)		22.00 (19.33-24.33)		16.67 (14.00-19.00)		18.67 (16.67-20.67)	
**Orientation (measured by scores); CHARLS *P*<.001, MHAS *P*<.001**									
	Mean (SD)	4.00 (1.14)		4.71 (0.57)		2.40 (0.88)		2.68 (0.66)	
	Median (IQR; quartiles 1-3)	4.00 (3.00-5.00)		5.00 (5.00-5.00)		3.00 (2.00-3.00)		3.00 (3.00-3.00)	
**Immediate verbal recall (measured by scores); CHARLS *P*<.001, MHAS *P*<.001**									
	Mean (SD)	4.12 (2.00)		5.82 (1.52)		4.54 (1.21)		5.19 (1.16)	
	Median (IQR; quartiles 1-3)	4.00 (3.00-5.67)		6.00 (5.00-7.00)		4.67 (3.67-5.33)		5.33 (4.33-6.00)	
**Delayed verbal recall (measured by scores); CHARLS *P*<.001, MHAS *P*<.001**									
	Mean (SD)	4.13 (2.60)		6.38 (1.83)		4.00 (1.99)		4.75 (1.84)	
	Median (IQR; quartiles 1-3)	4.00 (2.00-6.00)		6.00 (5.00-8.00)		4.00 (3.00-5.00)		5.00 (4.00-6.00)	
**Serial 7s (measured by scores); CHARLS *P*<.001**									
	Mean (SD)	2.87 (1.88)		3.92 (1.54)		—^d^		—	
	Median (IQR; quartiles 1-3)	3.00 (1.00-5.00)		5.00 (3.00-5.00)		—		—	
**Figure recall (measured by scores); CHARLS *P*<.001, MHAS *P*<.001**									
	Mean (SD)	0.49 (0.53)		0.71 (0.51)		5.33 (1.25)		5.73 (0.81)	
	Median (IQR; quartiles 1-3)	0.00 (0.00-1.00)		1.00 (0.00-1.00)		6.00 (5.00-6.00)		6.00 (5.00-6.00)	

^a^CHARLS: China Health and Retirement Longitudinal Study.

^b^MHAS: Mexican Health & Aging Study.

^c^*P* values are derived from the Wilcoxon rank-sum test.

^d^Not applicable.

### Association Between Digital Exclusion and Cognitive Scores Throughout the Follow-Up Waves

**Figure 2:**
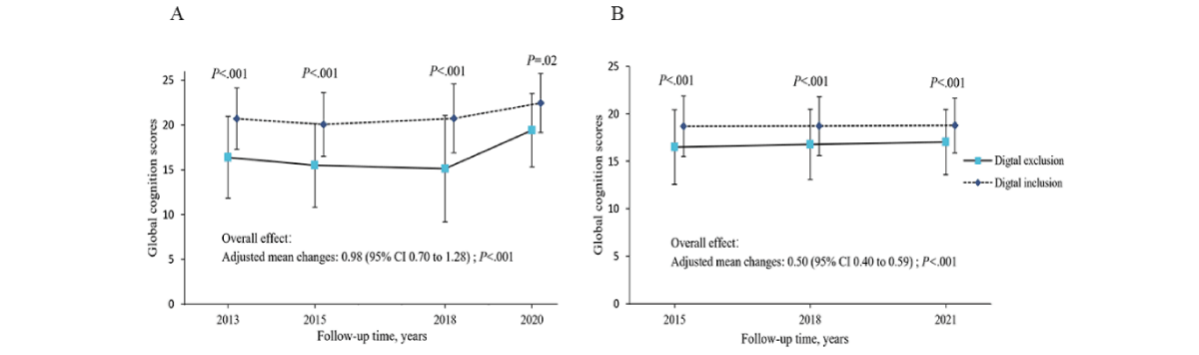
Average follow-up global cognitive scores in (A) CHARLS and (B) MHAS. Values are adjusted for baseline global cognitive scores, age, gender, BMI group, marital status, education level, residence, smoking status, BADL disability, IADL disability, and chronic diseases. BADL: basic activities of daily living; CHARLS: China Health and Retirement Longitudinal Study; IADL: instrumental activities of daily living; MHAS: Mexican Health & Aging Study.

**Figure 3:**
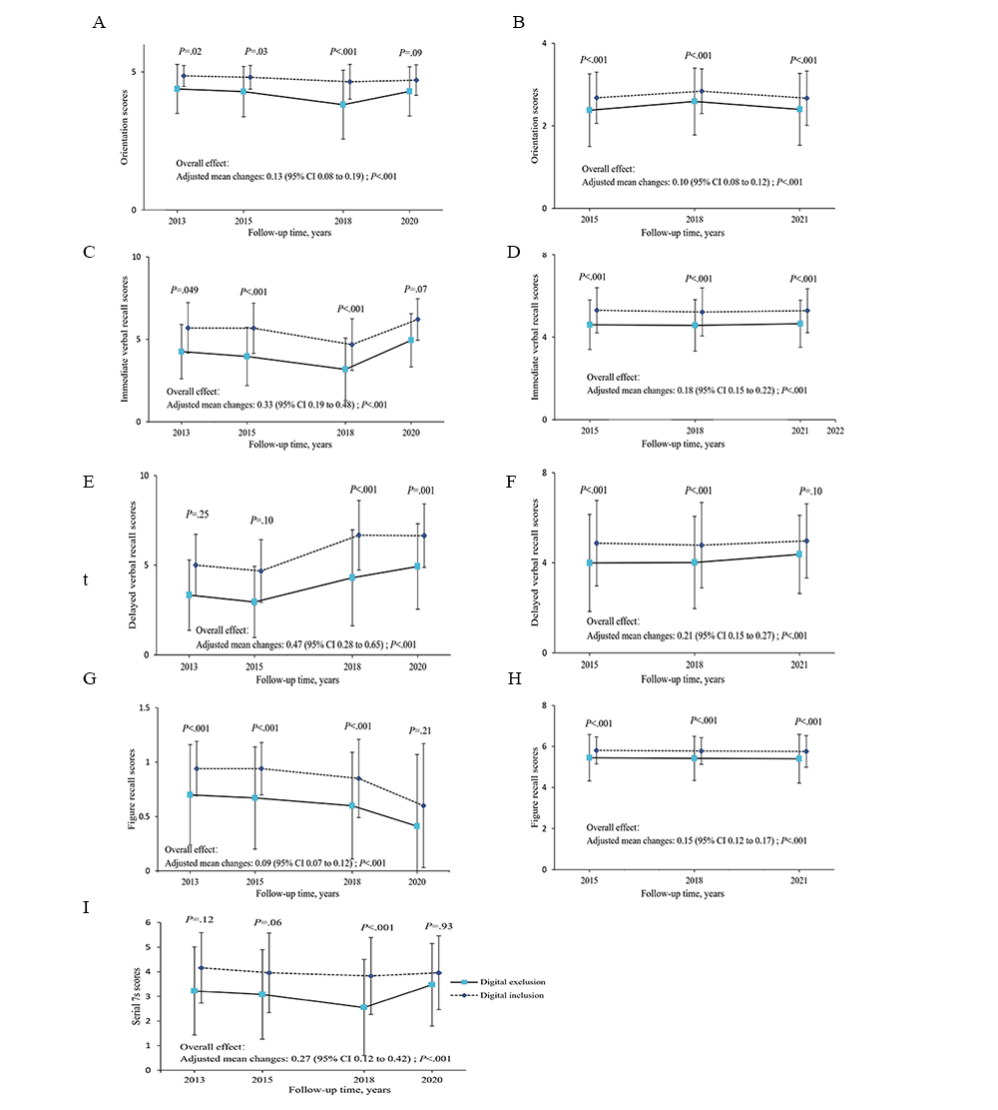
Average follow-up tests’ cognitive scores in CHARLS and MHAS. (A) Orientation scores in CHARLS, (B) orientation scores in MHAS, (C) immediate verbal recall scores in CHARLS, (D) immediate verbal recall scores in MHAS, (E) delayed verbal recall scores in CHARLS, (F) delayed verbal recall scores in MHAS, (G) figure recall scores in CHARLS, (H) figure recall scores in MHAS, and (I) serial 7s scores in CHARLS. Values are adjusted for baseline cognitive scores, age, gender, BMI group, marital status, educational level, residence, smoking status, BADL and IADL disabilities, and chronic diseases. BADL: basic activities of daily living; CHARLS: China Health and Retirement Longitudinal Study; IADL: instrumental activities of daily living; MHAS: Mexican Health & Aging Study.

In analyzing the cognitive score performance, participants experiencing digital exclusion had lower global cognitive scores than those experiencing digital inclusion. The overall difference in global cognitive scores was 0.98 (95% CI 0.70-1.28) in CHARLS and 0.50 (95% CI 0.40-0.59) in MHAS, as detailed in Tables S8 and S9 in Multimedia Appendix 1, respectively, and Figure 2. GEE models indicated that participants in the digital exclusion group were significantly more likely to have lower cognitive scores during follow-up periods (2013-2020 in CHARLS and 2015-2021 in MHAS), albeit with marginal differences (Tables S8 and S9 in Multimedia Appendix 1, respectively). Overall group differences in individual components of the expanded composite outcome were largely consistent. These components included orientation (CHARLS: 0.13, 95% CI 0.09-0.19; MHAS: 0.10, 95% CI 0.08-0.12), immediate verbal recall (CHARLS: 0.33, 95% CI 0.19-0.48; MHAS: 0.18, 95% CI 0.15-0.22), delayed verbal recall (CHARLS: 0.47, 95% CI 0.28-0.65; MHAS: 0.21, 95% CI 0.15-0.27), serial 7s (CHARLS: 0.27, 95% CI 0.12-0.42), and figure recall (CHARLS: 0.09, 95% CI 0.07-0.12; MHAS: 0.15, 95% CI 0.12-0.17), as shown in Figure 3 and Tables S8 and S9 in Multimedia Appendix 1, respectively.

## Discussion

### Principal Findings

As the prevalence of cognitive dysfunction in older populations increases, with no effective medical treatment available to cure or delay its progression, addressing modifiable risk factors becomes crucial. Considering that up to 40% of dementia cases may be attributable to modifiable risk factors [41], identifying interventions to slow cognitive decline, a potential precursor to dementia, is imperative. Our study examined the longitudinal relationship between digital exclusion and cognitive performance among 11,505 middle-aged and elderly Chinese participants and 12,560 older adults in Mexico. Digital exclusion rates ranged from 96.78% in China and 70.02% in Mexico. We observed that the elderly experiencing digital exclusion were more likely to exhibit worse cognitive function compared to others, and this association persisted even after adjusting for potential covariates and was found to be independent of gender and age.

Cognitive assessments are crucial for identifying cognitive impairment. The Mini-Mental State Exam (MMSE), which is widely used, has shown limited sensitivity in detecting mild cognitive impairment (MCI) [42]. Conversely, the Montreal Cognitive Assessment (MoCA) is a more effective tool for early MCI detection, incorporating tasks such as memory recall, delayed recall, and visuospatial and language tasks [42]. However, MoCA scores are significantly influenced by the individual’s education level [43]. Our study, informed by prior research [28,29,44], evaluated cognition across multiple tests, including memory, executive functioning, visuospatial abilities, and orientation. Generally, the cognitive function of the 2 groups was evaluated based on the orientation test, the immediate/delayed verbal recall test, and the figure recall test. The serial 7s test was not included in MHAS. Due to the diverse cultural backgrounds of the 2 countries, the verbal recall test set different words and total scores in the 2 cohorts, and a similar situation was found in the figure recall test. After standardizing cognitive test scores to a range of 0-1 and distinguishing between higher and lower cognitive scores using a threshold of 0.5, we found that the score distribution of the same type of cognitive test in CHARLS and MHAS is inconsistent. (Figure S3 in Multimedia Appendix 1). Although the differing distribution of cognitive scores across the CHARLS and MHAS waves provides a unique opportunity to generalize our findings across cultures, it also highlights the challenge of maintaining uniformity in test application across diverse cohorts. For future research, standardizing cognitive assessments across varied cohorts will be crucial.

In MHAS, we observed that although the cognitive scores of the digital inclusion group were generally higher than those of the digital exclusion group (follow-up waves 4-6, 2015-2021), there appeared to be no significant difference in cognitive scores among different waves (follow-up waves 4-6, 2015-2021). Through the questionnaire study, we consider that this consistency may be attributed to the uniformity of assessment questions across different waves, which guarantees the stability of assessment results. Repeatedly conducting the same test inevitably leads to the formation of memories, although it remains uncertain whether time can erase those memories. We observed a trend of declining cognitive scores in the tests of orientation, immediate verbal recall, serial 7s, and figure recall in CHARLS (follow-up waves 2-4, 2013-2018). To capture the nuances of cognition, various word lists were used to evaluate immediate and delayed verbal recall in the follow-up waves of CHARLS. In wave 5 (2020), words more aligned with Chinese cultural contexts were introduced, such as substituting “queen” with “president.” Additionally, wave 5 (2020) considered dialect tolerability, leading to higher scores in verbally oriented tasks. These methodological adjustments resulted in increased scores in orientation, word recall, and serial 7s in wave 5 compared to earlier waves. Despite these changes, significant disparities in cognitive scores persisted between digitally inclusive and exclusive groups. The use of varied assessment techniques indirectly enhanced the robustness of our findings, underscoring the impact of digital exclusion on poor cognitive performance.

Research in developing countries across Eastern and Western cultures indicates that older individuals facing digital exclusion are at a heightened risk of cognitive decline. This association is especially marked in China, where digital exclusion rates are notably higher. Analysis of CHARLS reveals a progressive widening of the cognitive score gap between digitally excluded and included individuals, particularly evident from follow-up waves 2-4 (Figure 2). In contrast, this gap remained stable across similar periods in MHAS. The observed heterogeneity between cohorts may stem from lower education levels in CHARLS compared to MHAS (85.28% vs 78.78% with lower secondary education) and a higher prevalence of comorbidities, such as arthritis or rheumatism, which may impede functional exercise and social engagement, exacerbating cognitive decline. Further investigation is warranted to explore how digital exclusion may accelerate cognitive decline among the elderly, especially across varying levels of exclusion.

#### Underlying Mechanisms

The mechanisms linking digital exclusion to diminished cognitive performance are not fully understood, yet several hypotheses have been proposed. One explanation involves the activation of brain regions during internet text reading, such as the left inferior frontal, temporal, posterior cingulate, parietal, and occipital regions, which are associated with language, reading, memory, and visual abilities [45]. Online cognitive function training, particularly for elderly patients at high risk of cognitive decline or dementia, has been shown to enhance cognitive scores, including memory and executive functions, more effectively than traditional face-to-face training [46]. Additionally, internet usage, which often involves searching activities, engages more neural circuitry compared to mere reading of text pages, especially in internet-savvy older adults (aged 55-76 years) [47]. Another interesting avenue of research is the study of astrocytes in mice, which showed that their calcium activity gradually increases when adapting to a new environment, thereby enhancing cognitive ability [48]. Investigating whether a similar process occurs in individuals newly introduced to the internet could provide further insights into the relationship between digital inclusion and cognitive performance. Digital exclusion intensifies the disparities in medical resource distribution, consequently hastening cognitive decline among the elderly [49]. For instance, patients with impaired vestibular function can benefit from online training programs designed for recovery [50,51]. However, elderly individuals who are unfamiliar with or uncomfortable using digital technology face significant barriers in accessing these online medical services. Compounding this, conditions such as hearing loss can further hinder the effectiveness of these digital interventions, potentially accelerating cognitive decline [52]. Moreover, limited internet access restricts opportunities for elderly individuals to make friends online, further isolating them and exacerbating cognitive deterioration [53]. However, internet users are generally more engaged in social activities, which can reduce social loneliness and enhance cognitive functions. To address the imbalanced distribution of medical resources caused by the digital divide among the elderly, the Chinese government has implemented “integrated medical services and elderly care” (IMSEC). These include the Home IMSEC model, the Community IMSEC model, the Institutional IMSEC model, and the Internet Plus IMSEC model [54]. The Japanese government has proposed a similar policy [55].

### Strengths and Limitations

Previous studies have often been limited to cross-sectional analyses focusing on a single follow-up wave [23]. In contrast, our study leveraged longitudinal cohorts to deepen understanding. In MHAS, we assessed cognitive differences between populations affected by digital exclusion and those with data inclusion across 3 follow-up waves. Meanwhile, CHARLS provided 4 waves of follow-up data. Although existing studies have established a link between digital exclusion and cognitive function, our longitudinal approach further bolsters the argument for the relationship in the elderly population. Furthermore, prior research has typically concentrated on single-country analyses [15-17,24,25]. Our study, however, enhanced the generalizability of these findings, using a cross-cultural, longitudinal design with individual-level cohorts from 2 developing countries, China and Mexico. Our study also built on the existing literature by incorporating more follow-up waves and broadening the geographic scope, thus offering a deeper understanding of digital exclusion’s impact on cognitive health in older populations. Additionally, the use of GEE models, which account for correlations among multiple waves of longitudinal data, reduces the potential for misestimation. Furthermore, compared to other studies [24,25], our research not only examined the longitudinal changes in participants’ cognitive scores over the years but also performed a horizontal comparison of population cognitive function using *Z*-score standardization. It not only considered the overall cognitive level of the population but also combined its own changes, which makes our research conclusions more reliable. Overall, our study expanded upon previous research findings by incorporating additional follow-up waves and broadening the geographical scope of the study areas, thereby offering a more comprehensive understanding of the impact of digital exclusion on cognitive health in older individuals.

The findings of our study were subject to several limitations. First, the assessment of digital exclusion was based on self-reported data. Despite the data collectors being well trained, there remains a potential for information bias due to variations in patients’ willingness to provide accurate answers. This variability could have unknown impacts on the observed associations, potentially skewing the results. Second, longitudinal data frequently suffer from incompleteness. Despite efforts to select a representative sample, not all respondents participated in the entire follow-up waves. In CHARLS, 10.47% of the participants were observed for a period of 2 years, 18.61% for 4 years, 30.33% for 7 years, and 40.59% for 9 years. Similarly, in MHAS, 15.45% were followed for 3 years, 18.10% for 6 years, and 66.46% for 9 years. Although previous research has indicated that attrition in cohort studies involving older individuals may not necessarily imply bias, this remains a consideration [56]. Third, although the GEE provides unbiased population-averaged regression coefficients [57], this analysis did not establish a causal relationship between internet use and cognitive abilities, nor did it rule out the possibility of reverse causality, where lower cognitive function might reduce internet use among older adults. Fourth, our study only enrolled 2 cohorts from developing countries. Therefore, the generalizability of our ﬁndings to other developing nations or regions with different socioeconomic and cultural contexts needs further evaluation. Although our study adjusted for common factors known to affect cognition—including baseline cognitive level, age, gender, education, marital status, lifestyle, chronic diseases, and basic daily living abilities—there remains the possibility of residual or unmeasured confounding. Factors such as genetic predisposition, cognitive reserve, or additional lifestyle variables could still influence the results. Additionally, our study’s design does not permit the establishment of a causal relationship between internet use and cognitive abilities. Internet use is also relevant in terms of social connections and a sense of belonging in society, which in turn could affect cognition. Thus, there is also the potential for reverse causality, as lower cognitive function may decrease the likelihood of internet use among older adults. To clarify these associations, further research involving the design of studies that use instrumental variable analyses may be necessary to rigorously test and potentially demonstrate a causal relationship.

### Conclusion

The findings of our study suggest that internet access may be associated with a reduced risk of cognitive deterioration in the elderly, highlighting the potential benefits of tailored digital inclusion strategies to promote active aging. However, there is a need for more robust evidence to substantiate the positive effects of digital inclusion in older adults. Future research should include interventional trials expanded across a broader range of countries and regions. Such studies are essential to develop customized strategies that effectively bridge the digital divide, thereby potentially enhancing cognitive health and overall well-being in aging populations.

## Appendix

Multimedia Appendix 1 Supplementary materials.

## Abbreviations

aGSIF: adjusted generalized standard error inflation factor
BADL: basic activities of daily living
CHARLS: China Health and Retirement Longitudinal Study
GEE: generalized estimating equation
GVIF: generalized variance inflation factor
IADL: instrumental activities of daily living
IMSEC: integrated medical services and elderly care
MCI: mild cognitive impairment
MHAS: Mexican Health & Aging Study
MoCA: Montreal Cognitive Assessment
OR: odds ratio

## Data Availability

The China Health and Retirement Longitudinal Study and the Mexican Health & Aging Study data are available online upon request [26,27]. The source codes are available from the corresponding author.
