# Supplementary material for: Digital Exclusion and Cognitive Function in Elderly Populations in Developing Countries: Insights Derived From 2 Longitudinal Cohort Studies
Source: J Med Internet Res. 2024 Nov 15;26:e56636. doi: 10.2196/56636 (PMC11607572; doi:10.2196/56636)
Supplement: Multimedia Appendix 1 [file jmir_v26i1e56636_app1.docx]

**Supplementary Materials**

[**Table S1.** Details of cognitive scores in CHARLS. 2](#_Toc171267043)

[**Table S2.** Details of cognitive scores in MHAS. 6](#_Toc171267044)

[**Table S3.** Details of covariates in CHARLS. 8](#_Toc171267045)

[**Table S4.** Details of covariates in MHAS 11](#_Toc171267046)

[**Table S5.** Descriptive characteristics of socio-demographic, and health-related information stratified by exposure. 14](#_Toc171267047)

[**Table S6.** Association of digital exclusion and worse cognition. 17](#_Toc171267048)

[**Table S7**. GVIF of confounding factors in analyses model of CHARLS and MHAS data. 18](#_Toc171267049)

[**Table S8**. Generalized estimating equation model to identify associations between digital exclusion and cognitive scores in CHARLS. 19](#_Toc171267050)

[**Table S9.** Generalized estimating equation model to identify associations between digital exclusion and cognitive score in MHAS. 21](#_Toc171267051)

[**Figure S1**. Flowcharts of sample selection. 22](#_Toc171267052)

[**Figure S2**. Pareto chart with missing covariates in CHARLS and MHAS. 23](#_Toc171267053)

[**Figure S3**. Cognitive scores distribution during follow-up waves in CHARLS and MHAS. 24](#_Toc171267054)

# **Table S1.** Details of cognitive scores in CHARLS.

| **Variables** | **Item numbers in the questionnaire** | **Questionnaire items and corresponding scores** | **Definitions** |
| --- | --- | --- | --- |
| Wave 1 (2011) | | | |
| Orientation | DC001 | Please tell me today’s date. (3, 2, 1, 0) | **1=**Year is correct / Month is correct / Day is correct  **0=**NA |
|  | DC002 | Please tell me the day of the week. Is it Monday, Tuesday, Wednesday, Thursday, Friday, Saturday, or Sunday? (1, 0) | **1=**Day of week OK/correct  **0=**Day of week not OK/incorrect |
|  | DC003 | What is the current season (among Spring, Summer, Fall, or Winter)? (1, 0) | **1=**Season OK  **0**=Season not OK |
| Immediate verbal recall | DC007 | Circle all the words mentioned by the R on the column. (10, 9, 8, 7, 6, 5, 4, 3, 2, 1, 0) | **1**=Rice / River / Doctor / Clothes / Egg / Cat / Bowl / Child / Hand / Book  **1**=Stool / Foot / Sky / Money / Pillow / Dog / House / House / School / Tea  **1=**Mountain / Stone / Blood / Mother / Shoes / Eye / Girl / House / Road / Sun  **1**=Water / Hospital / Tree / Father / Fire / Tooth / Moon / Village / Boy / Table  **0=**NA / None recalled |
| Delayed verbal recall | DC027 | A little while ago, I read you a list of words and you repeated the ones you could remember. Please tell me any of the words that you remember now. (10, 9, 8, 7, 6, 5, 4, 3, 2, 1, 0) | **1**=Rice / River / Doctor / Clothes / Egg / Cat / Bowl / Child / Hand / Book  **1**=Stool / Foot / Sky / Money / Pillow / Dog / House / House / School / Tea  **1=**Mountain / Stone / Blood / Mother / Shoes / Eye / Girl / House / Road / Sun  **1**=Water / Hospital / Tree / Father / Fire / Tooth / Moon / Village / Boy / Table  **0=**NA / None recalled |
| Serial 7s | DC019 | Let's try some subtraction of numbers this time. What does 100 minus 7 equal? (1, 0) | **1**=93  **0**=Others |
|  | DC020 | And 7 from that? (1, 0) | **1**=86  **0**=Others |
|  | DC021 | And 7 from that? (1, 0) | **1=**79  **0=**Others |
|  | DC021 | And 7 from that? (1, 0) | **1=**72  **0=**Others |
|  | DC023 | And 7 from that? (1, 0) | **1=**65  **0**=Others |
| Figure recall | DC025 | Do you see this picture? Please draw that picture on this paper. (1, 0) | **1=**Drew the picture  **0=**Failed to draw the picture |
| Wave 2 (2013) | | | |
| Orientation | DC001 | Please tell me today’s date (3, 2, 1, 0) | **1**=Year is correct / Month is correct / Day is correct  **0**=NA |
|  | DC002 | Please tell me the day of the week. Is it Monday, Tuesday, Wednesday, Thursday, Friday, Saturday, or Sunday? (1, 0) | **1**=Day of week OK/correct  **0**=Day of week not OK/incorrect |
|  | DC003 | What is the current season (among Spring, Summer, Fall, or Winter)? (1, 0) | **1**=Season OK  **0**=Season not OK |
| Immediate verbal recall | DC006 | Try to remember the words I just read to you. I’ll ask you to recall them later (10, 9, 8, 7, 6, 5, 4, 3, 2, 1, 0) | **1**=DC006_Wordlist [1] / DC006_Wordlist [2] / DC006_Wordlis t[3] / DC006_Wordlis [4] / DC006_Wordlist [5] / DC006_Wordlist [6] / DC006_Wordlist [7] / DC006_Wordlist [8] / DC006_Wordlis t[9] / DC006_Wordlist [10]  0=NA |
| Delayed verbal recall | DC027 | A little while ago, I read you a list of words and you repeated the ones you could remember. Please tell me any of the words that you remember now (10, 9, 8, 7, 6, 5, 4, 3, 2, 1, 0) | **1**=DC006_Wordlist [1] / DC006_Wordlist [2] / DC006_Wordlis t[3] / DC006_Wordlis [4] / DC006_Wordlist [5] / DC006_Wordlist [6] / DC006_Wordlist [7] / DC006_Wordlist [8] / DC006_Wordlis t[9] / DC006_Wordlist [10]  **0**=NA |
| Serial 7s | DC019 | Let's try some subtraction of numbers this time. What does 100 minus 7 equal? (1, 0) | **1=**93  **0=**Others |
|  | DC020 | And 7 from that? (1, 0) | **1**=86  **0=**Others |
|  | DC021 | And 7 from that? (1, 0) | **1**=79  **0**=Others |
|  | DC021 | And 7 from that? (1, 0) | **1**=72  **0**=Others |
|  | DC023 | And 7 from that? (1, 0) | **1**=65  **0=**Others |
| Figure recall | DC025 | Do you see this picture? Please draw that picture on this paper. (1, 0) | **1**=Drew the picture  **0**=Failed to draw the picture |
| Wave 3 (2015) | | | |
| Orientation | DC001 | Please tell me today’s date. (3, 2, 1, 0) | **1**=Year is correct / Month is correct / Day is correct  **0**=NA |
|  | DC002 | Please tell me the day of the week. Is it Monday, Tuesday, Wednesday, Thursday, Friday, Saturday, or Sunday? (1, 0) | **1**=Day of week OK/correct  **0=**Day of week not OK/incorrect |
|  | DC003 | What is the current season (among Spring, Summer, Fall, or Winter)? (1, 0) | **1=**Season OK  **0**=Season not OK |
| Immediate verbal recall | DC006 | Please tell me any of the words that you remember now. (10, 9, 8, 7, 6, 5, 4, 3, 2, 1, 0) | **1**=DC006_Wordlist [1] / DC006_Wordlist [2] / DC006_Wordlis t[3] / DC006_Wordlis [4] / DC006_Wordlist [5] / DC006_Wordlist [6] / DC006_Wordlist [7] / DC006_Wordlist [8] / DC006_Wordlis t[9] / DC006_Wordlist [10]  **0**=NA |
| Delayed verbal recall | DC027 | A little while ago, I read you a list of words and you repeated the ones you could remember. Please tell me any of the words that you remember now. (10, 9, 8, 7, 6, 5, 4, 3, 2, 1, 0) | **1**=DC006_Wordlist [1] / DC006_Wordlist [2] / DC006_Wordlis t[3] / DC006_Wordlis [4] / DC006_Wordlist [5] / DC006_Wordlist [6] / DC006_Wordlist [7] / DC006_Wordlist [8] / DC006_Wordlis t[9] / DC006_Wordlist [10]  **0**=NA |
| Serial 7s | DC019 | Let's try some subtraction of numbers this time. What does 100 minus 7 equal? (1, 0) | **1**=93  **0**=Others |
|  | DC020 | And 7 from that? (1, 0) | **1=**86  **0**=Others |
|  | DC021 | And 7 from that? (1, 0) | **1=**79  **0**=Others |
|  | DC021 | And 7 from that? (1, 0) | **1**=72  **0**=Others |
|  | DC023 | And 7 from that? (1, 0) | **1**=65  **0**=Others |
| Figure recall | DC025 | Do you see this picture? Please draw that picture on this paper. (1, 0) | **1**=Drew the picture  **0=**Failed to draw the picture |
| Wave 4 (2018) | | | |
| Orientation | DC001_W4 | What is the year? (1, 0) | **1=**Correct  **0=**Error |
|  | DC006_W4 | What is the month? (1, 0) |  |
|  | DC003_W4 | What is the date? (1, 0) |  |
|  | DC005_W4 | What is the day of the week? (1, 0) |  |
|  | DC002_W4 | What is the season of the year? (1, 0) |  |
| Immediate verbal recall | DC028_W4 | Now please tell me the words you can recall. (10, 9, 8, 7, 6, 5, 4, 3, 2, 1, 0) | **1=**Butter / Arm / Shore / Letter / Queen / Cabin / Pole / Ticket / Grass / Engine  **0=**0 / None |
|  | DC029_W4 |  |  |
|  | DC030_W4 |  |  |
| Delayed verbal recall | DC047_W4 | Please select words that is being correctly recalled by respondent. (10, 9, 8, 7, 6, 5, 4, 3, 2, 1, 0) | **1**=Butter / Arm / Shore / Letter / Queen / Cabin / Pole / Ticket / Grass / Engine  **0**=0 / None |
| Serial 7s | DC014_W4_1 | Record answer from respondent. (1, 0) | **1=**93  **0**=Others |
|  | DC014_W4_2 | Record answer from respondent. (1, 0) | **1**=86  **0**=Others |
|  | DC014_W4_3 | Record answer from respondent. (1, 0) | **1=**79  **0**=Others |
|  | DC014_W4_4 | Record answer from respondent. (1, 0) | **1**=72  **0**=Others |
|  | DC014_W4_5 | Record answer from respondent. (1, 0) | **1**=65  **0**=Others |
| Figure recall | DC024_W4 | Here is a drawing. Please copy the drawing on this paper. (1, 0) | **1**=Correct  **0**=Error |
| Wave 5 (2020) | | | |
| Orientation | DC001 | What is the year? (1, 0) | **1**=Correct  **0**=Error |
|  | DC005 | What is the month? (1, 0) |  |
|  | DC003 | What is the date? (1, 0) |  |
|  | DC004 | What is the day of the week? (1, 0) |  |
|  | DC002 | What is the season of the year? (1, 0) |  |
| Immediate verbal recall | DC012 | Now please tell me the words you can recall. (10, 9, 8, 7, 6, 5, 4, 3, 2, 1, 0) | **1**=Wordlist [1] / Wordlist [2] / Wordlist [3] / Wordlist [4] / Wordlist [5] / Wordlist [6] /Wordlist [7] / Wordlist [8] / Wordlist [9] / Wordlist [10]  **0**=0 |
|  | DC013 |  |  |
|  | DC014 |  |  |
| Delayed verbal recall | DC028 | Please select words that is being correctly recalled by respondent. (10, 9, 8, 7, 6, 5, 4, 3, 2, 1, 0) | **1=**Wordlist [1] / Wordlist [2] / Wordlist [3] / Wordlist [4] / Wordlist [5] / Wordlist [6] /Wordlist [7] / Wordlist [8] / Wordlist [9] / Wordlist [10]t  **0**=0 |
| Serial 7s | DC007_1 | Record answer from respondent. (1, 0) | **1**=93  **0**=Others / Do not know |
|  | DC007_2 | Record answer from respondent. (1, 0) | **1**=86  **0**=Others / Do not know |
|  | DC007_3 | Record answer from respondent. (1, 0) | **1**=79  **0**=Others / Do not know |
|  | DC007_4 | Record answer from respondent. (1, 0) | **1**=72  **0**=Others / Do not know |
|  | DC007_5 | Record answer from respondent. (1, 0) | **1**=65  **0**=Others / Do not know |
| Figure recall | DC009 | Here is a drawing. Please copy the drawing on this paper. (1, 0) | **1**=Correct  **0**=Error |

# **Table S2.** Details of cognitive scores in MHAS.

| **Variables** | **Item numbers in the questionnaire** | **Questionnaire items and corresponding scores** | **Definitions** |
| --- | --- | --- | --- |
| Wave 1 (2012) | | | |
| Orientation | E.11a | Did the informant correctly identify the year? (1, 0) | **1**=Yes  **0**=No / DK |
|  | E.11b | Did the informant correctly identify the month? (1, 0) |  |
|  | E.11c | Did the informant correctly identify the day? (1, 0) |  |
| Immediate verbal recall | E.7a | I am going to read a list of words. Listen carefully. When I have finished, you must repeat all the words you can. The order does not matter (8, 7, 6, 5, 4, 3, 2, 1, 0) | **1**=Cat / Arm / Pear / Lime / Chair / House / Mouse / Skirt  **1=**Dog / Hand / Bed / Strawberry / Boot / Lemon / Table / Cow  **0**=0 |
|  | E.7b |  |  |
|  | E.7c |  |  |
| Delayed verbal recall | E.14 | Do you remember the long list of words that I read before? Please tell me all the words of the list that you can remember, in whatever order (8, 7, 6, 5, 4, 3, 2, 1, 0) | **1**=Cat / Arm / Pear / Lime / Chair / House / Mouse / Skirt  **1=**Dog / Hand / Bed / Strawberry / Boot / Lemon / Table / Cow  **0**=0 |
| Figure recall | E.8 | Draw this picture in the space below. Try to draw the picture in order to make it exactly as mine. I will indicate count the time. I will indicate to you when you can start and when you can stop. (6, 5, 4, 3, 2, 1, 0) | Continuous variable=6, 5, 4, 3, 2, 1, 0 |
| Wave 2 (2015) | | | |
| Orientation | E.11a | Can you tell me which day is today? (Day, Month, Year) (3, 2, 1, 0) | **1**=Yes  **0=**No / DK |
|  | E.11b |  |  |
|  | E.11c |  |  |
| Immediate verbal recall | E.7a | I am going to read a list of words. Listen carefully. When I have finished, you must repeat all the words you can. The order does not matter. (8, 7, 6, 5, 4, 3, 2, 1, 0) | **1**=Cat / Arm / Pear / Lime / Chair / House / Mouse / Skirt  **1=**Dog / Hand / Bed / Strawberry / Boot / Lemon / Table / Cow  **0**=0 |
|  | E.7b |  |  |
|  | E.7c |  |  |
| Delayed verbal recall | E.14 | Do you remember the long list of words that I read before? Please tell me all the words of the list that you can remember, in whatever order. (8, 7, 6, 5, 4, 3, 2, 1, 0) | **1**=Cat / Arm / Pear / Lime / Chair / House / Mouse / Skirt  **1=**Dog / Hand / Bed / Strawberry / Boot / Lemon / Table / Cow  **0**=0 |
| Figure recall | E.8 | Draw this picture in the space below. Try to draw the picture in order to make it exactly as mine. I will indicate count the time. I will indicate to you when you can start and when you can stop. (6, 5, 4, 3, 2, 1, 0) | Continuous scores=6, 5, 4, 3, 2, 1, 0 |
| Wave 3 (2018) | | | |
| Orientation | E.11a | Can you tell me which day is today? (Day, Month, Year) (3, 2, 1, 0) | **1=**Yes  **0=**No / DK |
|  | E.11b |  |  |
|  | E.11c |  |  |
| Immediate verbal recall | E.7_1 | I am going to read a list of words. listen carefully. when I have finished reading, you must repeat all the words you can. the order does not matter. are you ready? (8, 7, 6, 5, 4, 3, 2, 1, 0) | **1**=Cat / Arm / Pear / Lime / Chair / House / Mouse / Skirt  **1=**Dog / Hand / Bed / Strawberry / Boot / Lemon / Table / Cow  **0**=0 |
|  | E.7_2 |  |  |
|  | E.7_3 |  |  |
| Delayed verbal recall | E.14 | Do you remember the long list of words that I read before? Please tell me all the words of the list that you can remember, in whatever order. (8, 7, 6, 5, 4, 3, 2, 1, 0) | **1**=Cat / Arm / Pear / Lime / Chair / House / Mouse / Skirt  **1=**Dog / Hand / Bed / Strawberry / Boot / Lemon / Table / Cow  **0**=0 |
| Figure recall | E.8 | Draw this picture in the space below. Try to draw the picture in order to make it exactly as mine. I will indicate count the time. I will indicate to you when you can start and when you can stop. (6, 5, 4, 3, 2, 1, 0) | Continuous scores=6, 5, 4, 3, 2, 1, 0 |
| Wave 4 (2021) | | | |
| Orientation | E.11a | Can you tell me which day is today? Tell me the day, month and year. (3, 2 , 1, 0) | **1=**Yes  **0**=No / D |
|  | E.11a |  |  |
|  | E.11a |  |  |
| Immediate verbal recall | E.7_1 | I am going to read a list of words. Listen carefully. When I have finished reading, you must repeat all the words you can. The order does not matter. Are you ready? (8, 7, 6, 5, 4, 3, 2, 1, 0) | **1**=Cat / Arm / Pear / Lime / Chair / House / Mouse / Skirt  **1=**Dog / Hand / Bed / Strawberry / Boot / Lemon / Table / Cow  **0**=0 |
|  | E.7_2 |  |  |
|  | E.7_3 |  |  |
| Delayed verbal recall | E.14 | Do you remember the long list of words that I read before? Please tell me all the words of the list that you can remember, in whatever order. (8, 7, 6, 5, 4, 3, 2, 1, 0) | **1**=Cat / Arm / Pear / Lime / Chair / House / Mouse / Skirt  **1=**Dog / Hand / Bed / Strawberry / Boot / Lemon / Table / Cow  **0**=0 |
| Figure recall | E.8 | Draw this picture in the space below. Try to draw the picture in order to make it exactly as mine. I will indicate count the time. I will indicate to you when you can start and when you can stop. (6, 5, 4, 3, 2, 1, 0) | Continuous scores=6, 5, 4, 3, 2, 1, 0 |

# **Table S3.** Details of covariates in CHARLS.

| **Covariates** | **Item numbers in the questionnaire** | **Questionnaire items and definitions** | **Responses transformation** |
| --- | --- | --- | --- |
| Demographic characteristic | | | |
| Age | BA002(Wave 1 - 2) | When were you born? | Continuous variable;  Categorical variable=<50; 50-59; >59 |
|  | BA004(Wave 1) | What is your age? |  |
|  | BA002 (Wave 3 - 4) | What’s your actual date of birth? |  |
|  | BA004_W3(Wave 3 - 4) | What’s your date of birth on ID card or Household register? |  |
| Gender | CV005 (Wave 1) | Record gender of main respondent. (Male, Female) | **Male=**Male  **Female=**Female |
|  | BA000_W2_3(Wave 2 - 4) | Interviewer record R’s gender. (Male, Female) |  |
| Body Mass Index ^*^ | PL002(Wave 1) | Record weight measurement. | Continuous variable  Categorical variable=>24; ≤24 |
|  | QL002(Wave 1) | Record height measurement. |  |
| Marital status | BE001(Wave 1) | What is your marital status? (Single, Married or cohabiting, Divorced or widowed) | **Single**=Never married  **Married or cohabiting**=Married with spouse present; Married but not living with spouse temporarily for reasons such as work  **Divorced or widowed**=Separated; Divorced; Widowed |
| Educational level | BD001(Wave 1) | What is the highest level of education you have attained? (Lower secondary, Upper secondary and vocational training, Tertiary) | **Lower secondary**=No formal education; Did not finish primary school but capable of reading and/or writing; Sishu / home school; Elementary school; Middle school  **Upper secondary and vocational training=**High school; Vocational school  **Tertiary**=Two- / Three-Year College / Associate degree; Four-Year College / Bachelor’s degree; Master’s degree; Doctoral degree / Ph.D. |
| Residence | A001(Wave 1) | Take down the type of this neighbourhood. (Rural, Urban) | **Rural=**Rural Village  **Urban**=Urban Community |
| Living type | | | |
| Smoking status | DA061(Wave 1) | Do you still have the habit or have you totally quit? (Ever, Current) | **Ever**=Quit  **Current=**Still have |
|  | DA059(Wave 1- 4) | Have you ever chewed tobacco, smoked a pipe, smoked self-rolled cigarettes, or smoked cigarettes/cigars? (Never) | **Never**=No |
| Drinking status | DA067(Wave 1) | Did you drink any alcoholic beverages, such as beer, wine, or liquor in the past year? How often? (Current, Never) | **Current=**Drink more than once a month; Drink but less than once a month  **Never=**None of these |
|  | DA069(Wave 1- 4) | Did you ever drink alcoholic beverages in the past? How often? (Never) | **Never=**I never had a drink |
| Chronic disease | | | |
| Hypertension | DA007(Wave 1) | Have you been diagnosed with hypertension by a doctor? (Yes, No) | **Yes=**Yes  **No**=No |
| Diabetes |  | Have you been diagnosed with diabetes or high blood sugar by a doctor? (Yes, No) |  |
| Dyslipidemia |  | Have you been diagnosed with dyslipidemia by a doctor? (Yes, No) |  |
| Heart disease |  | Have you been diagnosed with heart attack, coronary heart disease, angina, congestive heart failure, or other heart problems by a doctor? (Yes, No) |  |
| Respiratory illness |  | Have you been diagnosed with chronic lung diseases / asthma by a doctor? (Yes, No) |  |
| Liver disease |  | Have you been diagnosed with liver disease by a doctor? (Yes, No) |  |
| Kidney disease |  | Have you been diagnosed with kidney disease (except for tumor or cancer) by a doctor? (Yes, No) |  |
| Digestive disease |  | Have you been diagnosed with stomach or other digestive disease (except for tumor or cancer) by a doctor? (Yes, No) |  |
| Arthritis or rheumatism |  | Have you been diagnosed with arthritis or rheumatism by a doctor? (Yes, No) |  |
| Cancer |  | Have you been diagnosed with cancer or malignant tumor (excluding minor skin cancers) by a doctor? (Yes, No) |  |
| Basic activities of daily living | | | |
| Continence | DB003(Wave 1) | Do you have difficulty walking 1 km? (1,0) | **1=**No, I don’t have any difficulty; I have difficulty but can still do it.  **0=**Yes, I have difficulty and need help; I can not do it. |
| Dressing | DB010(Wave 1) | Do you have any difficulty with dressing? (1,0) |  |
| Bathing | DB011(Wave 1) | Do you have any difficulty with bathing or showering? (1,0) |  |
| Feeding | DB012(Wave 1) | Do you have any difficulty with eating? (1,0) |  |
| Transferring | DB013(Wave 1) | Do you have any difficulty with getting into or out of bed? (1,0) |  |
| Going to toilet | DB014(Wave 1) | Do you have any difficulties with using the toilet? (1,0) |  |
| Disability basic activities of daily living | DB003, DB010, DB011, DB012, DB03, DB014 (Wave 1) | Do you have difficulty walking 1 km; Do you have any difficulty with dressing; Do you have any difficulty with bathing or showering; Do you have any difficulty with bathing or showering; Do you have any difficulty with eating; Do you have any difficulties with using the toilet? (1,0) | **1=**(any of basic activities of daily living scores=0)  **0**=(sum of basic activities of daily living scores=5) |
| Instrumental activities of daily living | | | |
| Housekeeping | DB016(Wave 1) | Because of health and memory problems, do you have any difficulties with doing household chores? (1,0) | **1=**No, I don’t have any difficulty; I have difficulty but can still do it.  **0=**Yes, I have difficulty and need help; I can not do it. |
| Preparing hot meals | DB017(Wave 1) | Because of health and memory problems, do you have any difficulties with preparing hot meals? (1,0) |  |
| Shopping | DB018(Wave 1) | Because of health and memory problems, do you have any difficulties with shopping for groceries? By shopping, we mean deciding what to buy and paying for it. (1,0) |  |
| Managing money | DB019(Wave 1) | Because of health and memory problems, do you have any difficulties with managing your money, such as paying your bills, keeping track of expenses, or managing assets? (1,0) |  |
| Taking medications | DB020(Wave 1) | Because of health and memory problems, do you have any difficulties with taking medications? By taking medications, we mean taking the right portion of medication right on time? (1,0) |  |
| Disability instrumental activities of daily living | DB016; DB017; DB018; DB019; DB020 (Wave 1) | Because of health and memory problems, do you have any difficulties with doing household chores; Because of health and memory problems, do you have any difficulties with preparing hot meals; Because of health and memory problems, do you have any difficulties with shopping for groceries? By shopping, we mean deciding what to buy and paying for it; Because of health and memory problems, do you have any difficulties with managing your money, such as paying your bills, keeping track of expenses, or managing assets; Because of health and memory problems, do you have any difficulties with taking medications? By taking medications, we mean taking the right portion of medication right on time? (1,0) | **1=**(any of instrumental activities of daily living scores=0)  **0=**(sum of instrumental activities of daily living scores=5) |
| Anthropometric Measures | | | |
| Hand grip test ^b^ | QC003(Wave 1) | First record of left hand grip strength | Continuous variable |
|  | QC004(Wave 1) | First record of right hand grip strength |  |
|  | QC005(Wave 1) | Second record of left hand grip strength |  |
|  | QC006(Wave 1) | Second record of right hand grip strength |  |
| Waist circumference | QM002(Wave 1) | Record waist measurements | Continuous variable |

^a^ Body Mass Index = weight (kg) / height(m)^2^

^b^ Hand grip test comes from the average strength of the two hands.

# **Table S4.** Details of covariates in MHAS

| **Covariates** | **Item numbers in the questionnaire** | **Questionnaire items and definitions** | **Responses transformation** |
| --- | --- | --- | --- |
| **Demographic characteristic** | | | |
| Age | A.1 (Wave 1) | On what day, month, and year were you born? | Continuous variable;  Categorical variable=<50; 50-59; >59 |
|  | A.2 (Wave 2) | How old are you in fill years? |  |
|  | A.2b (Wave 3 – 6) | How old are you in fill years? |  |
| Gender | sex (Wave 1) | Respondent’s sex | **Male=**Male  **Female=**Female |
|  | A.1 (Wave 2 – 6) | Indicate if the respondent is |  |
|  | AA.1 (Wave 22 – 6) | Indicate if the respondent is |  |
| Body Mass Index ^a^ | 1.7 (Wave 3) | Height | Continuous variable  Categorical variable=>24; ≤24 |
|  | 1.7.1(Wave 3) | To verify that I measured well, I will do it again. |  |
|  | 1.8(Wave 3) | Now I will measure your weight. |  |
|  | 1.8.1(Wave 3) | To verify that I measured well, I will do it again. |  |
| Marital status | A.3(Wave 3) | Currently are you single/married/in a civil union/divorced/separated from a civil union / separated from a marriage / widowed from a civil union / widowed from a marriage? (Single, Married or cohabiting, Divorced or widowed) | **Single**=single  **Married or cohabiting=**married; in a civil union  **Divorced or widowed**=divorced; separated from a civil union; separated from a marriage; widowed from a civil union; widowed from a marriage |
|  | AA.10(Wave 3) |  |  |
| Educational level | A.3 (Wave 1) | What is the last year or grade that you completed in school? (Lower secondary; Tertiary; Upper secondary and vocational training) | **Lower secondary**=None; Primary; Secondary  **Tertiary**=Technical or Commercial; Preparatory or High School; Basic teaching school  **Upper secondary and vocational training**=College; Graduate |
|  | AA.4a (Wave 2 – 6) |  |  |
| Residence | - | - | - |
| **Living type** | | | |
| Smoking status | C.51 (Wave 3) | Have you ever smoked cigarettes? (Ever, Current, Never) | **Ever**=(C.51=Yes & C.53=Yes & C.54=No); (C.51=Yes & C.54=No)；(C.51=Yes & C.53=No)  **Current=**(C.54=Yes)  **Never**=(C.51=No) |
|  | C.53 (Wave 3) | Have you smoked cigarettes in the last two years? (Ever, Current, Never) |  |
|  | C.54 (Wave 3) | Do you smoke cigarettes now?  (Ever, Current, Never) |  |
|  | C.51(wave4-6) | Have you ever smoked cigarettes? (Never) | **Never=**No |
| Drinking status | C.59a (Wave 3) | Currently, do you ever drink any alcoholic beverages such as beer, wine, liquor, or pulque (drink made from fermented cactus sap) (Drinking, Never) | **Drinking**=Yes  **Never=**No |
|  | C.59a (Wave 4-6) | Currently, do you ever drink any alcoholic beverages such as beer, wine, liquor, or pulque (drink made from fermented cactus sap) (Never) | **Never=**No |
| **Chronic disease** | | | |
| Hypertension | C.4(Wave 3) | Has a doctor or medical personnel ever diagnosed you with hypertension or high blood pressure? (Yes, No) | **Yes**=Yes  **No=**No |
| Diabetes | C.6(Wave 3) | Has a doctor or medical personnel ever diagnosed you with diabetes? (Yes, No) | **Yes=**Yes  **No=**No |
| Dyslipidemia | - | - | - |
| Heart disease | C.22a(Wave 3) | Has a doctor or medical personnel ever told you that you have had a heart attack? (Yes, No) | **Yes**=Yes  **No**=No |
| Respiratory illness | C.19(Wave 3) | Has a doctor or medical personnel ever told / diagnosed you with a respiratory illness, such as asthma or emphysema? (Yes, No) | **Yes**=Yes  **No**=No |
| Liver disease | - | - | - |
| Kidney disease | - | - | - |
| Digestive disease | - | - | - |
| Arthritis or rheumatism | C.32(Wave 3) | Has a doctor or medical personnel ever diagnosed you with arthritis or rheumatism? (Yes, No) | **Yes**=Yes  **No**=No |
| Cancer | C.12(Wave 3) | Has a doctor or medical personnel ever diagnosed you with cancer? (Yes, No) | **Yes=**Yes  **No**=No |
| **Basic activities of daily living** | | | |
| Continence | H.15(Wave 3) | Because of a health problem, do you have any difficulty walking across a room? (1,0) | **1**=No,  **0**=Yes; Can’t Do’’ |
| Dressing | H.13(Wave 3) | Because of a health problem, do you have difficulty including putting on shoes and socks? (1,0) |  |
| Bathing | H.16(Wave 3) | Because of a health problem, do you have any difficulty bathing or showering? (1,0) |  |
| Feeding | H.17(Wave 3) | Because of a health problem, do you have any difficulty eating, such as cutting your food? (1,0) |  |
| Transferring | H.18(Wave 3) | Because of a health problem, do you have any difficulty getting into or out of bed? (1,0) |  |
| Going to toilet | H.19(Wave 3) | Because of a health problem, do you have any difficulty using the toilet, including getting on and off the toilet or squatting? (1,0) |  |
| Basic activities of daily living difficulty | H.15; H.13; H.16; H.17; H.18; H.19 (Wave 3) | Because of a health problem, do you have any difficulty walking across a room; Because of a health problem, do you have difficulty including putting on shoes and socks; Because of a health problem, do you have any difficulty bathing or showering ; Because of a health problem, do you have any difficulty eating, such as cutting your food ; Because of a health problem, do you have any difficulty getting into or out of bed ; Because of a health problem, do you have any difficulty using the toilet, including getting on and off the toilet or squatting? (1,0) | **1=**(any of basic activities of daily living scores=0)  **0**=(sum of basic activities of daily living scores=6) |
| **Instrumental activities of daily living** | | | |
| Housekeeping | - | - | - |
| Preparing hot meals | H.26a(Wave 3) | Because of a health problem, do you have any difficulty preparing a hot meal? (1,0) | **1**=No,  **0**=Yes; Can't Do |
| Shopping | H.27a(Wave 3) | Because of a health problem, do you have any difficulty shopping for groceries? (1,0) |  |
| Managing money | H.29a(Wave 3) | Because of a health problem, do you have any difficulty managing your money? (1,0) |  |
| Taking medications | H.28a(Wave 3) | Because of a health problem, do you have any difficulty taking medications (if you take any or needed to do so)? (1,0) |  |
| Instrumental activities of daily living difficulty | H,26a; H.27a; H.29a; H.28a  (Wave 3) | Because of a health problem, do you have any difficulty preparing a hot meal; Because of a health problem, do you have any difficulty shopping for groceries; Because of a health problem, do you have any difficulty managing your money; Because of a health problem, do you have any difficulty taking medications (if you take any or needed to do so)? | **1=**(any of instrumental activities of daily living scores=0)  **0**=(sum of instrumental activities of daily living scores=4) |
| **Anthropometric Measures** | | | |
| Hand grip test ^b^ | 1.26(Wave 3) | We will do two measurements with the left hand. | Continuous variable |
|  | 1.27(Wave 3) | We will do two measurements with the left hand. |  |
| Waist circumference ^c^ | 1.9(Wave 3) | Waist | Continuous variable |
|  | 1.9.1(Wave 3) | To verify that I measured well, I will do it again. |  |

^a^ Body Mass Index = weight (kg) / height(m)^2^. Height and weight came from the average of the two measurements.

^b^ Hand grip test comes from the average strength of the two hands.

^c^ Waist circumference comes from the average of the average of the two measurements

# **Table S5.** Descriptive characteristics of socio-demographic, and health-related information stratified by exposure.

| **Variables** | **CHARLS**  **(N = 11505)** | | | **MHAS**  **(N =12560)** | | |
| --- | --- | --- | --- | --- | --- | --- |
|  | **Digital exclusion**  **(N=11135)** | **Digital inclusion**  **(N=370)** | ***P-value*** | **Digital exclusion**  **(N=8794)** | **Digital inclusion**  **(N=3766)** | ***P-value*** |
| Age (years), mean (SD) | 57.43 (8.75) | 52.70 (7.59) | <.001 ^a^ | 63.25 (10.22) | 60.52 (9.23) | <.001 ^a^ |
| Gender, n (%) |  |  | .35 ^b^ |  |  | .08 ^b^ |
| Male | 5475 (49.17) | 191 (51.62) |  | 3575 (40.65) | 1595 (42.35) |  |
| Female | 5660 (50.83) | 179 (48.38) |  | 5219 (59.35) | 2171 (57.65) |  |
| Body mass index, kg/m^2^, mean (SD) | 23.91 (10.88) | 24.75 (4.07) | <.001 ^a^ | 28.80 (5.33) | 29.54 (5.09) | .003 ^a^ |
| Marital status, n (%) |  |  | .23 ^c^ |  |  | <.001 ^b^ |
| Single | 73 (0.66) | 1 (0.27) |  | 406 (4.62) | 148 (3.93) |  |
| Married or cohabiting | 10076 (90.49) | 345 (93.24) |  | 6157 (70.01) | 2953 (78.41) |  |
| Divorced or widowed | 986 (8.85) | 24 (6.49) |  | 2231 (25.37) | 665 (17.66) |  |
| Education, n (%) |  |  | <.001 ^c^ |  |  | <.001 ^b^ |
| Lower secondary | 9705 (87.16) | 106 (28.65) |  | 7916 (90.02) | 1979 (52.55) |  |
| Upper secondary and vocational training | 1230 (11.05) | 149 (40.27) |  | 610 (6.94) | 903 (23.98) |  |
| Tertiary | 196 (1.76) | 115 (31.08) |  | 232 (2.64) | 867 (23.02) |  |
| Unknown | 4 (0.04) | 0 (0.00) |  | 36 (0.41) | 17 (0.45) |  |
| Region of residence, n (%) |  |  | <.001 ^b^ |  |  | - |
| Rural | 5348 (48.03) | 26 (7.03) |  | - | - |  |
| Town | 3805 (34.17) | 301 (81.35) |  | - | - |  |
| Unknown | 1982 (17.80) | 43 (11.62) |  | - | - |  |
| Smoking status, n (%) |  |  | .29 ^c^ |  |  | <.001 ^c^ |
| Never | 6704 (60.21) | 240 (64.86) |  | 5762 (65.52) | 2240 (59.48) |  |
| Ever | 916 (8.23) | 29 (7.84) |  | 1989 (22.62) | 1016 (26.98) |  |
| Current | 3511 (31.53) | 101 (27.30) |  | 1042 (11.85) | 510 (13.54) |  |
| Unknown | 4 (0.04) | 0 (0.00) |  | 1 (0.01) | 0 (0.00) |  |
| Drinking, n (%) |  |  | <.001 ^c^ |  |  | <.001 ^c^ |
| Never | 7328 (65.81) | 198 (53.51) |  | 6852 (77.92) | 2645 (70.23) |  |
| Current | 3805 (34.17) | 172 (46.49) |  | 1939 (22.05) | 1121 (29.77) |  |
| Unknown | 2 (0.02) | 0 (0.00) |  | 3 (0.03) | 0 (0.00) |  |
| Chronic disease, n (%) |  |  |  |  |  |  |
| Hypertension | 2474 (22.22) | 86 (23.24) | .64 ^b^ | 3706 (42.14) | 1505 (39.96) | .06 ^b^ |
| Diabetes | 589 (5.29) | 19 (5.14) | .90 ^b^ | 1884 (21.42) | 746 (19.81) | .07 ^b^ |
| Dyslipidemia | 989 (8.88) | 72 (19.46) | <.001 ^b^ | - | - | - |
| Heart disease | 1285 (11.54) | 31 (8.38) | .06 ^b^ | 258 (2.93) | 113 (3.00) | .06 ^b^ |
| Respiratory illness | 1185 (10.64) | 18 (4.86) | <.001 ^b^ | 470 (5.34) | 191 (5.07) | .50 ^b^ |
| Liver disease | 442 (3.97) | 14 (3.78) | .86 ^b^ | - | - | - |
| Kidney disease | 692 (6.21) | 22 (5.95) | .83 ^b^ | - | - | - |
| Digestive disease | 2503 (22.48) | 64 (17.30) | .02 ^b^ | - | - | - |
| Arthritis or rheumatism | 3640 (32.69) | 56 (15.14) | <.001 ^b^ | 1200 (13.65) | 430 (11.42) | .002 ^b^ |
| Cancer | 99 (0.89) | 2 (0.54) | .77 ^b^ | 134 (1.52) | 95 (2.52) | <.001 ^b^ |
| Basic activities of daily living disability, n (%) |  |  | <.001 ^b^ |  |  | <.001 ^b^ |
| Yes | 667 (5.99) | 3 (0.81) |  | 1343 (15.27) | 357 (9.48) |  |
| No | 4595 (41.27) | 75 (20.27) |  | 3779 (42.97) | 1443 (38.32) |  |
| Unknow | 5873 (52.74) | 292 (78.92) |  | 3672 (41.76) | 1966 (52.20) |  |
| Instrumental activities of daily living disability, n (%) |  |  | <.001 ^c^ |  |  | <.001 ^c^ |
| Yes | 840 (7.54) | 4 (1.08) |  | 674 (7.66) | 170 (4.51) |  |
| No | 10289 (92.40) | 366 (98.92) |  | 7221 (82.11) | 3291 (87.39) |  |
| Unknow | 6 (0.05) | 0 (0.00) |  | 899 (10.22) | 305 (8.10) |  |
| Hand grip test, Kg |  |  | <.001 ^a^ |  |  | <.001 ^a^ |
| Mean (SD) | 30.80 (10.01) | 36.05 (10.41) |  | 24.71 (8.43) | 27.70 (8.75) |  |
| Median (IQR; quartiles 1-3) | 30.00 (23.50-37.50) | 34.50 (28.00-44.50) |  | 24.00 (19.00-30.00) | 26.00 (21.00-33.00) |  |
| Waist circumference, cm |  |  | .12 ^a^ |  |  | .08 ^a^ |
| Mean (SD) | 84.51 (12.56) | 85.04 (13.37) |  | 97.94 (12.58) | 99.01 (12.18) |  |
| Median (IQR; quartiles 1-3) | 84.90 (78.00-92.00) | 86.00 (79.10-93.00) |  | 97.07 (89.70-105.38) | 98.60 (90.26-106.50) |  |
| Baseline cognitive function ^b^ |  |  |  |  |  |  |
| Global cognitive scores |  |  | <.001 ^a^ |  |  | <.001 ^a^ |
| Mean (SD) | 16.38 (4.50) | 20.28 (3.51) |  | 17.15 (3.52) | 19.04 (2.90) |  |
| Median (IQR; quartiles 1-3) | 17.00 (13.00-20.00) | 21.00 (18.00-23.00) |  | 17.67 (15.00-19.67) | 19.33 (17.33-21.00) |  |
| Orientation scores |  |  | <.001 ^a^ |  |  | <.001 ^a^ |
| Mean (SD) | 4.30 (0.94) | 4.84 (0.40) |  | 2.46 (0.81) | 2.73 (0.57) |  |
| Median (IQR; quartiles 1-3) | 5.00 (4.00-5.00) | 5.00 (5.00-5.00) |  | 3.00 (2.00-3.00) | 3.00 (3.00-3.00) |  |
| Immediate verbal recall scores |  |  | <.001 ^a^ |  |  | <.001 ^a^ |
| Mean (SD) | 4.25 (1.63) | 5.63 (1.43) |  | 4.68 (1.16) | 5.34 (1.09) |  |
| Median (IQR; quartiles 1-3) | 4.00 (3.00-5.00) | 6.00 (5.00-7.00) |  | 4.67 (4.00-5.33) | 5.33 (4.67-6.00) |  |
| Delayed verbal recall scores |  |  | <.001 ^a^ |  |  | <.001 ^a^ |
| Mean (SD) | 3.32 (1.92) | 4.74 (1.80) |  | 4.37 (2.01) | 5.13 (1.79) |  |
| Median (IQR; quartiles 1-3) | 3.00 (2.00-5.00) | 5.00 (4.00-6.00) |  | 5.00 (3.00-6.00) | 5.00 (4.00-6.00) |  |
| Serial 7s scores |  |  | <.001 ^a^ |  |  | - |
| Mean (SD) | 3.27 (1.82) | 4.15 (1.44) |  | - | - |  |
| Median (IQR; quartiles 1-3) | 4.00 (1.00-5.00) | 5.00 (4.00-5.00) |  | - | - |  |
| Figure recall scores |  |  | <.001 ^a^ |  |  | <.001 ^a^ |
| Mean (SD) | 0.71 (0.45) | 0.92 (0.27) |  | 5.50 (1.08) | 5.79 (0.68) |  |
| Median (IQR; quartiles 1-3) | 1.00 (0.00-1.00) | 1.00 (1.00-1.00) |  | 6.00 (5.00-6.00) | 6.00 (6.00-6.00) |  |

*P-values* are derived from: a Wilcoxon rank-sum test, b Pearson’s Chi-squared test, c Fisher’s exact test.

The percentages have been rounded and may not total 100.

Abbreviations: SD: standard deviation; HDL: High-Density Lipoprotein Cholesterol; LDL: Low-Density Lipoprotein Cholesterol

# **Table S6.** Association of digital exclusion and worse cognition.

|  | **Digital exclusion** | | **Digital inclusion** | **Crude model** | | **Adjusted model** | |
| --- | --- | --- | --- | --- | --- | --- | --- |
|  | No. of worse cognition/Total (%) | | | OR (95% CI) | *P-value* | OR (95% CI) | *P-value* |
| **In CHARLS** | | | | | | | |
| Model 1 | 5819/11135  (52.26) | | 41/370  (11.08) | 8.78 (6.42-12.4) | <.001 | 3.37 (2.39-4.89) | <.001 |
| Model 2 |  |  |  |  |  | 2.05 (1.43-3.01) | <.001 |
| Model 3 |  |  |  |  |  | 2.06 (1.44-3.03) | <.001 |
| Model 4 |  |  |  |  |  | 2.04 (1.42-2.99) | <.001 |
| **In MHAS** | | | | | | | |
| Model 1 | 5371/8794  (61.08) | 1377/3766  (36.56) | | 2.72(2.52-2.95) | <.001 | 1.73 (1.57-1.90) | <.001 |
| Model 2 |  |  |  |  |  | 1.40 (1.26-1.55) | <.001 |
| Model 3 |  |  |  |  |  | 1.39 (1.25-1.54) | <.001 |
| Model 4 |  |  |  |  |  | 1.40 (1.26-1.55) | <.001 |

Model 1 was adjusted for age, gender, body mass index type and global cognitive scores in baseline. Model 2 was adjusted for marital status, educational level, residence, smoking status and drinking status based on Model 1. Model 3 was adjusted for basic activities of daily living disability and instrumental activities of daily living disability based on Model 2. Model 4 was adjusted chronic diseases on Model 3.

Abbreviations: OR: Odds Ratio, CI: Confidence Interval.

# **Table S7**. GVIF of confounding factors in analyses model of CHARLS and MHAS data.

| **Confounding Factors** | **CHARLS** | | | **MHAS** | | |
| --- | --- | --- | --- | --- | --- | --- |
|  | **GVIF** | **Df** | **aGSIF** | **GVIF** | **Df** | **aGSIF** |
| Age | 1.21 | 1 | 1.10 | 1.17 | 1 | 1.08 |
| Gender | 2.18 | 1 | 1.48 | 1.57 | 1 | 1.25 |
| Body Mass Index group | 1.15 | 2 | 1.04 | 1.02 | 2 | 1.01 |
| Marital status | 1.07 | 2 | 1.02 | 1.16 | 2 | 1.04 |
| Educational level | 1.12 | 3 | 1.02 | 1.25 | 3 | 1.04 |
| Residence | 1.12 | 2 | 1.03 | - | - | - |
| Smoking status | 1.96 | 3 | 1.12 | 2.56 | 3 | 1.17 |
| Drinking status | 1.35 | 1 | 1.16 | 2.37 | 2 | 1.24 |
| Basic activities of daily living difficulty | 1.28 | 2 | 1.06 | 1.35 | 2 | 1.08 |
| Instrumental activities of daily living difficulty | 1.11 | 1 | 1.06 | 1.21 | 2 | 1.05 |
| Hypertension | 1.18 | 1 | 1.09 | 1.15 | 2 | 1.04 |
| Dyslipidemia | 1.15 | 1 | 1.07 | - | - | - |
| Diabetes | 1.09 | 1 | 1.05 | 1.09 | 2 | 1.02 |
| Cancer | 1.01 | 1 | 1.00 | 1.19 | 2 | 1.04 |
| Liver disease | 1.03 | 1 | 1.01 | - | - | - |
| Heart disease | 1.15 | 1 | 1.07 | 1.09 | 2 | 1.02 |
| Kidney disease | 1.05 | 1 | 1.02 | - | - | - |
| Digestive disease | 1.08 | 1 | 1.04 | - | - | - |
| Arthritis or rheumatism | 1.11 | 1 | 1.05 | 1.10 | 2 | 1.02 |
| Respiratory illness | 1.07 | 1 | 1.03 | 1.27 | 2 | 1.06 |
| Baseline global cognitive scores | 1.06 | 1 | 1.03 | 1.06 | 1 | 1.03 |

Abbreviations: GVIF, generalized standard error inflation factor; Df, degree of freedom; aGSIF, adjusted generalized standard error inflation factor, aGSIF = GVIF^(1/(2*Df)).

# **Table S8**. Generalized estimating equation model to identify associations between digital exclusion and cognitive scores in CHARLS.

|  | **Digital exclusion**  **(N = 11155)** | **Digital inclusion**  **(N = 370)** | **Unadjusted**  **Mean changes***  **(95% CI)** | **Adjusted**  **Mean changes*** **(95% CI)** | ***P-value*** |
| --- | --- | --- | --- | --- | --- |
| **Global cognitive scores** | | | | | |
| Model effect ^a^  Overall effect, marginal mean | 16.30 (5.13) | 20.93 (3.63) | 4.31 (4.01-4.61) | 0.98 (0.70-1.28) | <.001 |
| Time |  |  |  |  | <.001 |
| Time × exposure interaction |  |  |  |  | <.001 |
| 2013, Mean (SD) | 16.39 (4.57) | 20.70 (3.42) | 4.05 (3.64-4.47) | 0.69 (0.28-1.11) | <.001 |
| 2015, Mean (SD) | 15.49 (4.66) | 20.06 (3.56) | 4.33 (3.91-4.76) | 0.97 (0.57-1.37) | <.001 |
| 2018, Mean (SD) | 15.12 (5.94) | 20.74 (3.85) | 5.12 (4.62-5.61) | 1.79 (1.31-2.27) | <.001 |
| 2020, Mean (SD) | 19.42(4.10) | 22.45(3.29) | 3.70 (3.28-4.12) | 0.49 (0.07-0.91) | .02 |
| **Orientation scores** | | | | | |
| Model effect ^a^  Overall effect, marginal mean | 4.19(1.04) | 4.76(0.51) | 0.54 (0.50-0.59) | 0.13 (0.08-0.19) | <.001 |
| Time |  |  |  |  | <.001 |
| Time × exposure interaction |  |  |  |  | <.001 |
| 2013, Mean (SD) | 4.39 (0.89) | 4.86 (0.38) | 0.49 (0.43-0.55) | 0.08 (0.01- 0.15) | .02 |
| 2015, Mean (SD) | 4.29 (0.91) | 4.81 (0.43) | 0.49 (0.42-0.55) | 0.08 (0.01-0.15) | .03 |
| 2018, Mean (SD) | 3.82 (1.25) | 4.65 (0.63) | 0.72 (0.63-0.81) | 0.29 (0.20-0.39) | <.001 |
| 2020, Mean (SD) | 4.30 (0.89) | 4.71 (0.55) | 0.47 (0.39-0.55) | 0.08 (-0.01-0.16) | .09 |
| **Immediate verbal recall scores** | | | | | |
| Model effect ^a^  Overall effect, marginal mean | 4.08 (1.83) | 5.57 (1.57) | 1.48 (1.32-1.64) | 0.33 (0.19-0.48) | <.001 |
| Time |  |  |  |  | <.001 |
| Time × exposure interaction |  |  |  |  | .003 |
| 2013, Mean (SD) | 4.25 (1.64) | 5.69 (1.53) | 1.36 (1.14-1.58) | 0.21 (0.00-0.43) | .049 |
| 2015, Mean (SD) | 3.96 (1.76) | 5.67 (1.52) | 1.58 (1.35-1.81) | 0.43 (0.23-0.64) | <.001 |
| 2018, Mean (SD) | 3.18 (1.89) | 4.68 (1.56) | 1.66 (1.43-1.90) | 0.52 (0.29-0.75) | <.001 |
| 2020, Mean (SD) | 4.94 (1.62) | 6.20 (1.26) | 1.31 (1.12-1.49) | 0.16 (-0.01-0.34) | .07 |
| **Delayed verbal recall scores** | | | | | |
| Model effect ^a^  Overall effect, marginal mean | 3.82 (2.38) | 5.72 (2.01) | 1.82 (1.63-2.01) | 0.47 (0.28-0.65) | <.001 |
| Time |  |  |  |  | <.001 |
| Time × exposure interaction |  |  |  |  | <.001 |
| 2013, Mean (SD) | 3.33 (1.97) | 5.01 (1.71) | 1.51 (1.25-1.76) | 0.15 (-0.11-0.42) | .25 |
| 2015, Mean (SD) | 2.95 (1.99) | 4.68 (1.75) | 1.57 (1.31-1.83) | 0.21 (-0.04-0.45) | .10 |
| 2018, Mean (SD) | 4.30 (2.68) | 6.66 (1.94) | 2.43 (2.15-2.72) | 1.08 (0.80-1.36) | <.001 |
| 2020, Mean (SD) | 4.93 (2.39) | 6.65 (1.77) | 1.77 (1.53-2.02) | 0.41 (0.16-0.66) | .001 |
| **Serial 7s scores** | | | | | |
| Model effect ^a^  Overall effect, marginal mean | 3.05 (1.85) | 3.98 (1.53) | 0.92 (0.79-1.06) | 0.27 (0.12-0.42) | <.001 |
| Time |  |  |  |  | <.001 |
| Time × exposure interaction |  |  |  |  | <.001 |
| 2013, Mean (SD) | 3.22 (1.79) | 4.16 (1.43) | 0.84 (0.62-1.05) | 0.18 (-0.05-0.40) | .12 |
| 2015, Mean (SD) | 3.08 (1.82) | 3.96 (1.62) | 0.87 (0.65-1.10) | 0.22 (-0.01-0.44) | .06 |
| 2018, Mean (SD) | 2.54 (1.95) | 3.82 (1.56) | 1.33 (1.12-1.55) | 0.67 (0.44-0.89) | <.001 |
| 2020, Mean (SD) | 3.47 (1.68) | 3.96 (1.50) | 0.62 (0.40-0.84) | -0.01 (-0.25-0.22) | .93 |
| **Figure recall scores** | | | | | |
| Model effect ^a^  Overall effect, marginal mean | 0.60 (0.53) | 0.84 (0.40) | 0.25 (0.23-0.28) | 0.09 (0.07-0.12) | <.001 |
| Time |  |  |  |  | <.001 |
| Time × exposure interaction |  |  |  |  | .09 |
| 2013, Mean (SD) | 0.70 (0.46) | 0.94 (0.25) | 0.24 (0.21-0.28) | 0.08 (0.04-0.12) | <.001 |
| 2015, Mean (SD) | 0.67 (0.47) | 0.94 (0.24) | 0.28 (0.25-0.31) | 0.12 (0.08-0.15) | <.001 |
| 2018, Mean (SD) | 0.60 (0.49) | 0.85 (0.36) | 0.29 (0.24-0.33) | 0.13 (0.08-0.18) | <.001 |
| 2020, Mean (SD) | 0.41 (0.66) | 0.60 (0.57) | 0.21 (0.13-0.28) | 0.05 (-0.03-0.12) | .21 |

^a^ Adjusted for baseline scores, age, gender, body mass index group, marital status, educational level, residence, smoking status, drinking status, basic activities of daily living difficulty, instrumental activities of daily living difficulty and chronic diseases.

# **Table S9.** Generalized estimating equation model to identify associations between digital exclusion and cognitive score in MHAS.

|  | **Digital exclusion** | **Digital inclusion** | **Unadjusted**  **Mean changes** * **(95% CI)** | **Adjusted**  **Mean changes** * **(95% CI)** | ***P-value*** |
| --- | --- | --- | --- | --- | --- |
| **Global cognitive scores** |  |  |  |  |  |
| Model effect ^a^  Overall effect, marginal mean | 16.74 (3.72) | 18.72 (3.08) | 1.97 (1.85-2.08) | 0.50 (0.40-0.59) | <.001 |
| Time |  |  |  |  | .26 |
| Time × exposure interaction |  |  |  |  | .005 |
| 2015, Mean (SD) | 16.49 (3.92) | 18.68 (3.18) | 2.10 (1.96-2.23) | 0.60 (0.48-0.71) | <.001 |
| 2018, Mean (SD) | 16.79 (3.70) | 18.71 (3.11) | 1.91 (1.77-2.06) | 0.47 (0.34-0.59) | <.001 |
| 2021, Mean (SD) | 17.03 (3.42) | 18.77 (2.90) | 1.83 (1.69-1.97) | 0.38 (0.26-0.51) | <.001 |
| **Orientation scores** |  |  |  |  |  |
| Model effect ^a^  Overall effect, marginal mean | 2.45 (0.86) | 2.73 (0.61) | 0.28 (0.26-0.31) | 0.10 (0.08-0.12) | <.001 |
| Time |  |  |  |  | <.001 |
| Time × exposure interaction |  |  |  |  | .005 |
| 2015, Mean (SD) | 2.38 (0.88) | 2.68 (0.62) | 0.31 (0.28-0.34) | 0.12 (0.09-0.15) | <.001 |
| 2018, Mean (SD) | 2.59 (0.81) | 2.84 (0.54) | 0.25 (0.22-0.28) | 0.07 (0.04-0.10) | <.001 |
| 2021, Mean (SD) | 2.40 (0.87) | 2.67 (0.66) | 0.29 (0.25-0.32) | 0.10 (0.07-0.14) | <.001 |
| **Immediate verbal recall scores** |  |  |  |  |  |
| Model effect ^a^  Overall effect, marginal mean | 4.60 (1.20) | 5.27 (1.12) | 0.66 (0.62-0.70) | 0.18 (0.15-0.22) | <.001 |
| Time |  |  |  |  | <.001 |
| Time × exposure interaction |  |  |  |  | .37 |
| 2015, Mean (SD) | 4.60 (1.20) | 5.30 (1.10) | 0.69 (0.64-0.73) | 0.20 (0.16-0.24) | <.001 |
| 2018, Mean (SD) | 4.57 (1.25) | 5.22 (1.17) | 0.65 (0.60-0.70) | 0.17 (0.13-0.22) | <.001 |
| 2021, Mean (SD) | 4.65 (1.14) | 5.28 (1.07) | 0.64 (0.59-0.69) | 0.17 (0.12-0.21) | <.001 |
| **Delayed verbal recall scores** |  |  |  |  |  |
| Model effect ^a^  Overall effect, marginal mean | 4.10 (2.02) | 4.87 (1.83) | 0.78 (0.72-0.84) | 0.21 (0.15-0.27) | <.001 |
| Time |  |  |  |  | <.001 |
| Time × exposure interaction |  |  |  |  | <.001 |
| 2015, Mean (SD) | 3.99 (2.16) | 4.87 (1.90) | 0.87 (0.79-0.95) | 0.29 (0.22-0.36) | <.001 |
| 2018, Mean (SD) | 4.01 (2.05) | 4.78 (1.90) | 0.77 (0.69-0.85) | 0.21 (0.13-0.29) | <.001 |
| 2021, Mean (SD) | 4.37 (1.74) | 4.97 (1.65) | 0.64 (0.57-0.72) | 0.09 (0.02-0.17) | .01 |
| **Figure recall scores** |  |  |  |  |  |
| Model effect ^a^  Overall effect, marginal mean | 5.43 (1.13) | 5.78 (0.69) | 0.34 (0.31-0.36) | 0.15 (0.12-0.17) | <.001 |
| Time |  |  |  |  | <.001 |
| Time × exposure interaction |  |  |  |  | .08 |
| 2015, Mean (SD) | 5.45 (1.13) | 5.81 (0.66) | 0.32 (0.29-0.35) | 0.12 (0.09-0.15) | <.001 |
| 2018, Mean (SD) | 5.42 (1.08) | 5.78 (0.65) | 0.34 (0.31-0.38) | 0.16 (0.12-0.19) | <.001 |
| 2021, Mean (SD) | 5.40 (1.19) | 5.76 (0.77) | 0.36 (0.31-0.40) | 0.17 (0.13-0.21) | <.001 |

^a^ Adjusted for baseline scores, age, gender, body mass index type, marital status, educational level, residence, smoking status, drinking status and chronic diseases.

# **Figure S1**. Flowcharts of sample selection.

1. Flowcharts of sample selection in CHARLS; (B) Flowcharts of sample selection in MHAS.


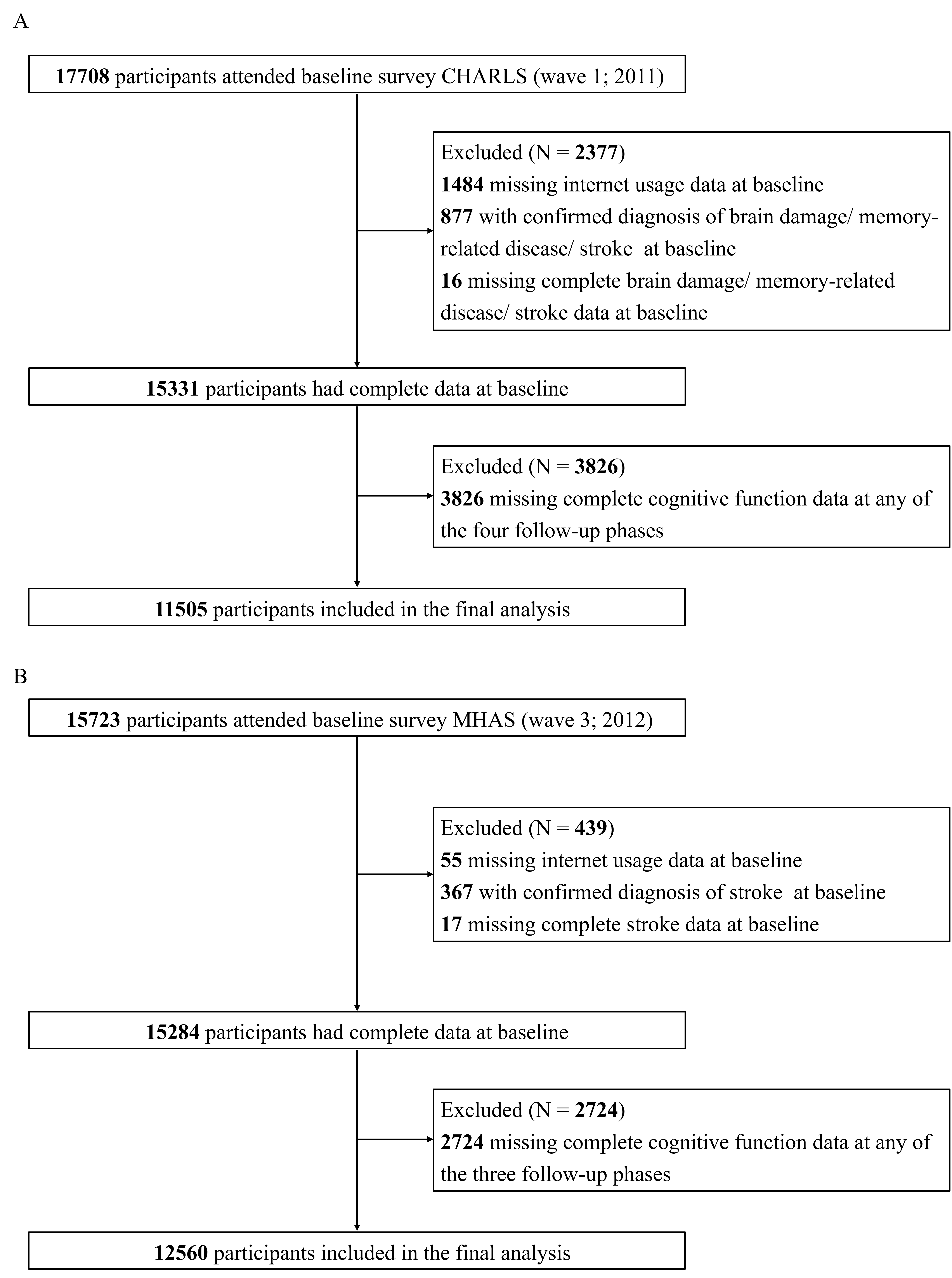


# **Figure S2**. Pareto chart with missing covariates in CHARLS and MHAS.


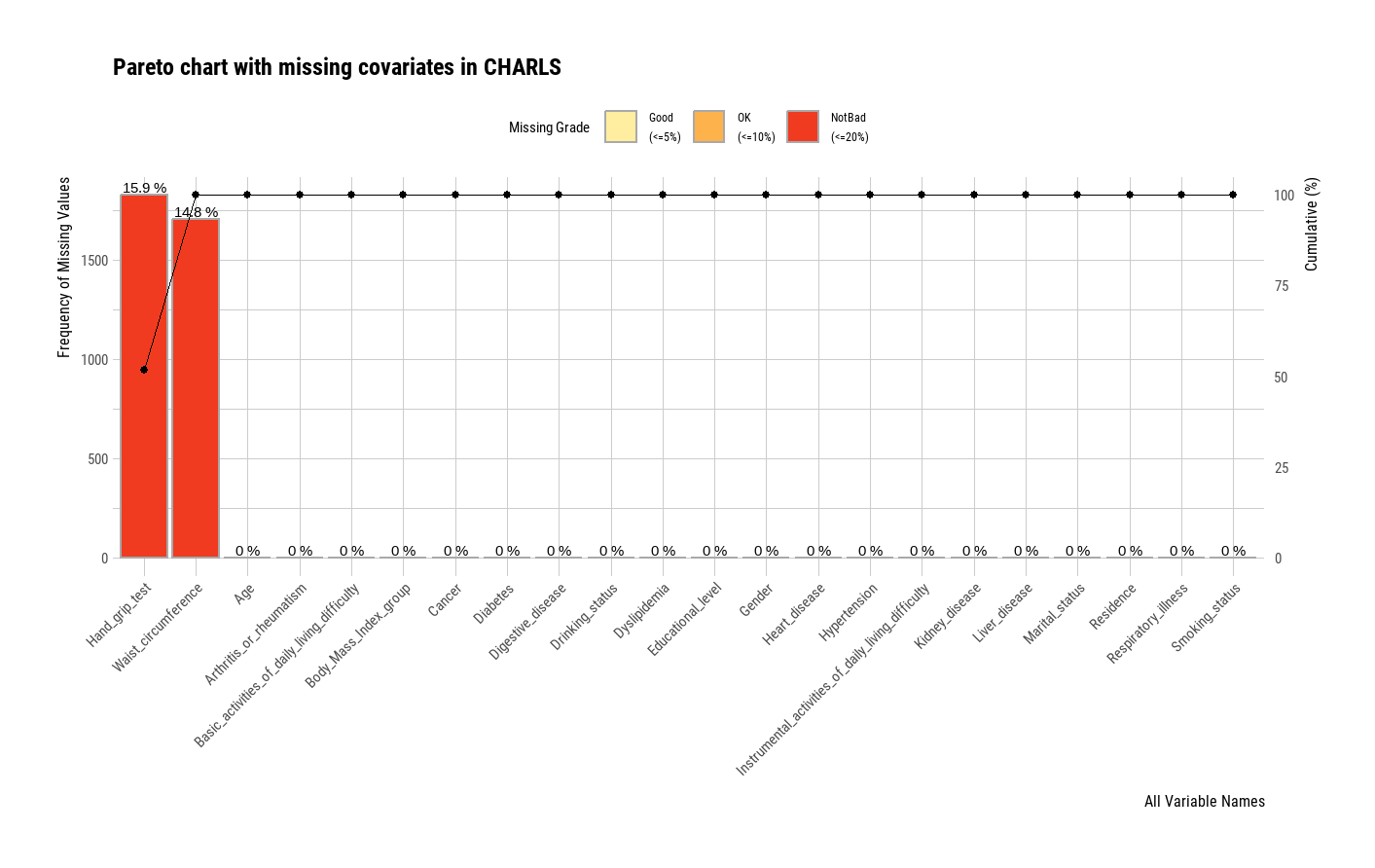

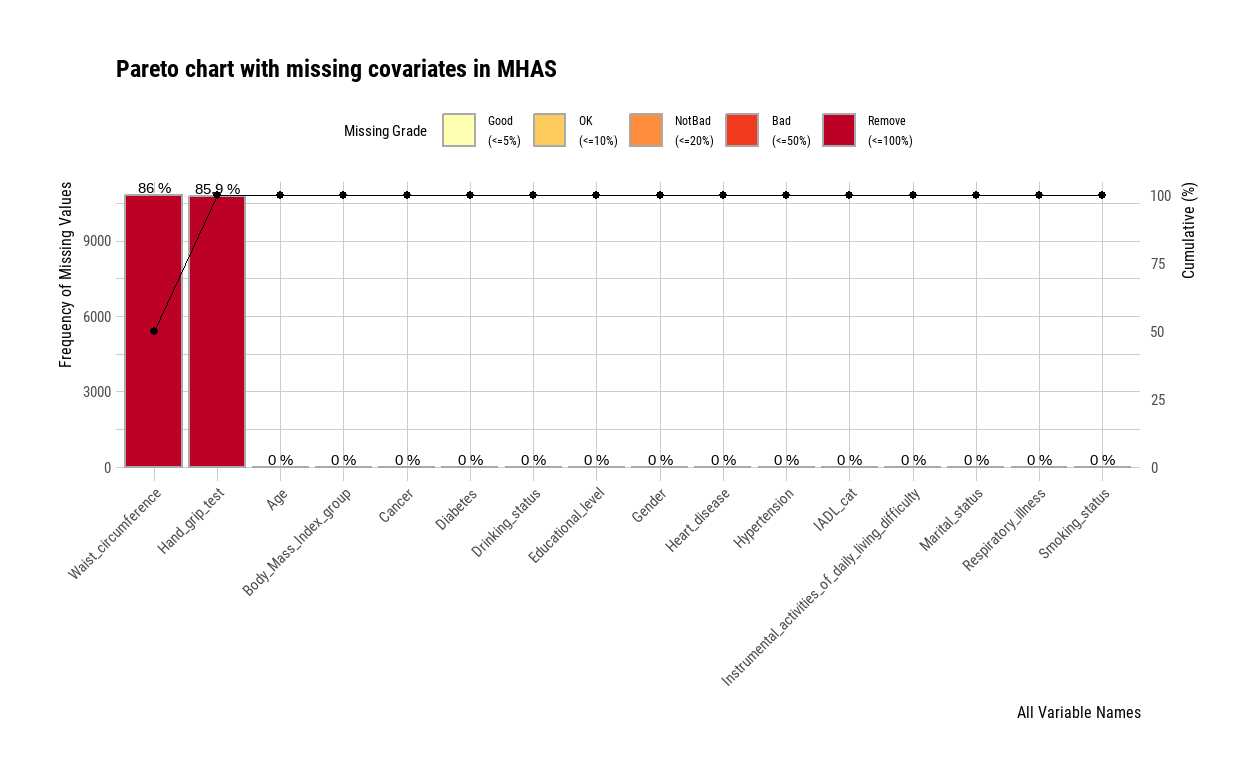


A

B

(A) Pareto chart with missing covariates in CHARLS; (B) Pareto chart with missing covariates in MHAS

# **Figure S3**. Cognitive scores distribution during follow-up waves in CHARLS and MHAS.


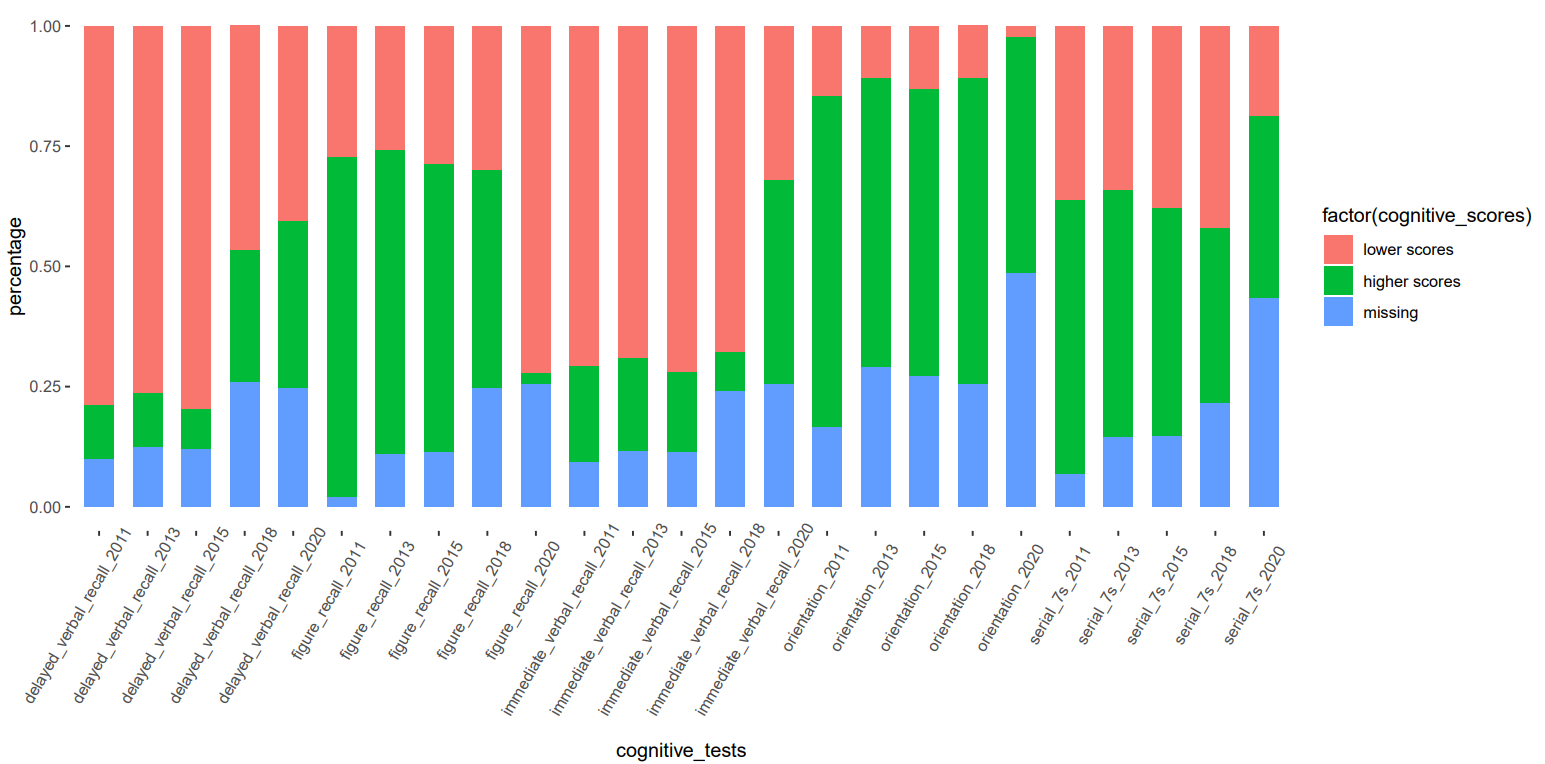

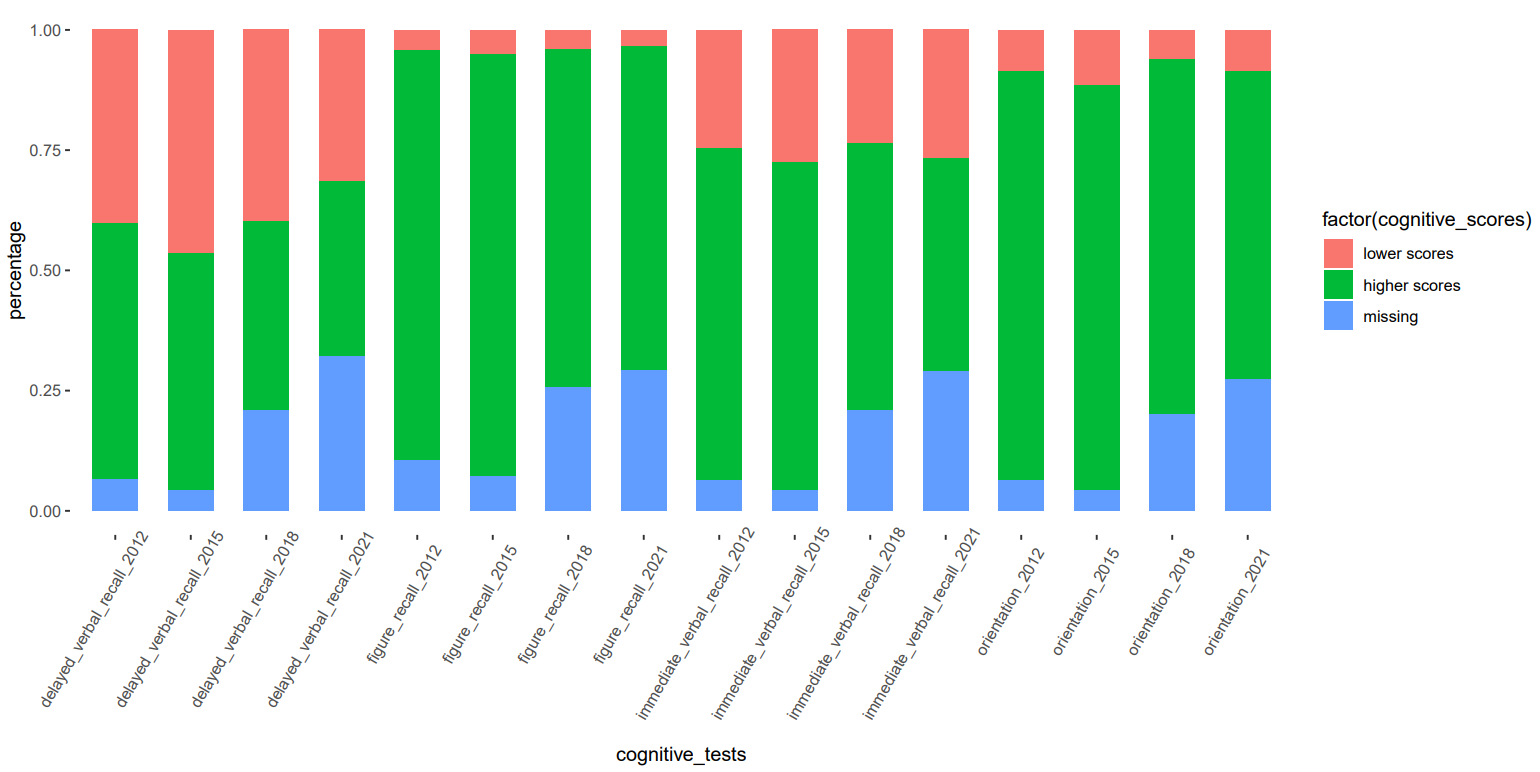


A

B

(A) Cognitive score distribution during follow-up waves in CHARLS; (B) Cognitive score distribution during follow-up waves in MHAS
